# A new azobenzene-based design strategy for detergents in membrane protein research†

**DOI:** 10.1039/d0sc01022g

**Published:** 2020-03-13

**Authors:** Leonhard H. Urner, Maiko Schulze, Yasmine B. Maier, Waldemar Hoffmann, Stephan Warnke, Idlir Liko, Kristin Folmert, Christian Manz, Carol V. Robinson, Rainer Haag, Kevin Pagel

**Affiliations:** Institute of Chemistry and Biochemistry, Freie Universität Berlin Arnimallee 22 14195 Berlin Germany leonhard.urner@fu-berlin.de; Department of Molecular Physics, Fritz Haber Institute of the Max Planck Society Faradayweg 4-6 14195 Berlin Germany kevin.pagel@fu-berlin.de; Physical and Theoretical Chemistry Laboratory, University of Oxford South Parks Road OX13QZ Oxford UK

## Abstract

Mass spectrometry enables the in-depth structural elucidation of membrane protein complexes, which is of great interest in structural biology and drug discovery. Recent breakthroughs in this field revealed the need for design rules that allow fine-tuning the properties of detergents in solution and gas phase. Desirable features include protein charge reduction, because it helps to preserve native features of protein complexes during transfer from solution into the vacuum of a mass spectrometer. Addressing this challenge, we here present the first systematic gas-phase study of azobenzene detergents. The utility of gas-phase techniques for monitoring light-driven changes of isomer ratios and molecular properties are investigated in detail. This leads to the first azobenzene detergent that enables the native mass spectrometry analysis of membrane proteins and whose charge-reducing properties can be tuned by irradiation with light. More broadly, the presented work outlines new avenues for the high-throughput characterization of supramolecular systems and opens a new design strategy for detergents in membrane protein research.

## Abstract 2

Here, L. H. Urner and co-workers identify a new detergent design strategy for the non-denaturing structural analysis of membrane proteins by studying the gas-phase properties of azobenzene-based oligoglycerol detergents.

## Graphical abstract

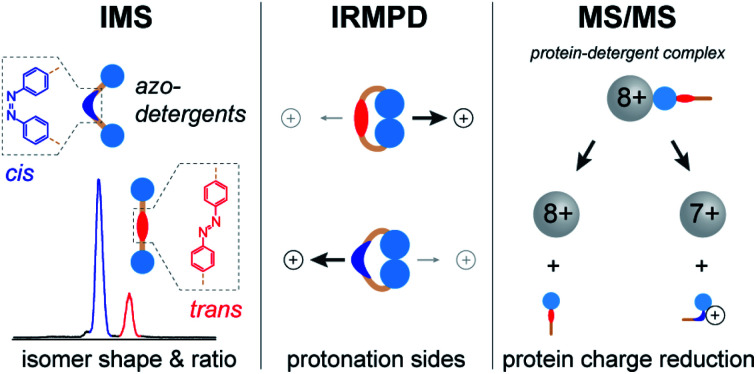

## Introduction

Proteins are molecular machines that are vital for living organisms. Approximately 30% of the proteome is associated with membranes. Membrane proteins account as targets for more than 50% of current drugs.^1^ Methods that help to elucidate their structure are therefore of great importance in structural biology and drug discovery. Besides their ubiquitous presence in daily life, detergents play a significant role in membrane protein research. A breakthrough came with the demonstration that detergent aggregates preserve non-covalent interactions of membrane protein complexes during transfer from solution into the vacuum of a mass spectrometer.^2^ Protein charge reduction has been identified as a key parameter in this process, because it lowers the disruptive impact of Coulomb interactions on protein structure.^3–5^ Protein charge reduction can be achieved in many ways, for example, by using basic solution additives,^3–5^ detergents,^4,6,7^ or by treating the electrospray plume with acetonitrile vapour.^3^ The extent of charge reduction that is needed for retaining native structural features of a protein complex is difficult to generalize and needs to be determined experimentally. However, charge-reducing additives and detergents provide often a more denaturing solution environment for proteins.^6,8^ Detergent designers are therefore faced with the challenge to identify design rules that allow to compromise the protein-compatibility of detergents in solution and their charge-reducing properties in the gas phase.^7^

Generally, detergents are of limited use when the intended application requires their properties to be dynamically altered during the application. To enable such remote control, detergents are often modified with azobenzene, which consists of two phenyl rings that are held together by a N=N bond.^9^ Azobenzene exists either in a thermodynamically more stable *trans* or a metastable *cis* form.^10^ The light-driven interconversion between both isomers effectively changes their structure,^10^ dipole,^11^ and basicity,^12,13^ which has driven the development of photoresponsive materials.^14–16^ Research on azobenzene detergents has been focused on photoresponsive surface coatings,^17–20^ catalysis systems,^21^ and supramolecular aggregates.^22–27^ The utility of anionic 4-hexylphenylazosulfonate for denaturing top-down proteomics has been outlined very recently.^28^ As ionic detergents commonly denature membrane proteins more than non-ionic detergents,^29^ this inspired us to investigate whether non-ionic azobenzene detergents can be used for the non-denaturing structural analysis of membrane proteins.

A ubiquitous challenge for structure–property analysis of azobenzene detergents is the characterization of their individual isomers. Condensed-phase techniques average the results obtained from the entire population of species within a sample. The light-driven isomerization is almost never quantitative^30^ rendering the unambiguous analysis of individual isomers difficult. Ion mobility-mass spectrometry (IM-MS) can circumvent this challenge, because it allows to characterize individual species in the presence of many others.^31^ Briefly, ions are separated according to the time required to pass a cell that is filled with a neutral buffer gas. This drift time depends on the mass, charge, and overall shape of an ion, which enables the separation of isomers.^24,32–35^ To gain further insights into the molecular structure, drift times are typically converted to collisional cross-section (CCS) values. They represent the ion's rotationally averaged cross section interacting with the buffer gas. CCS values are independent from instrumental parameters and can be derived from theoretical calculations.^36,37^ Comparing experimental and theoretical CCS values provides insights into the overall structure of isomers.^36^ While IM-MS is sensitive to the ions' overall shape, infrared multiple photon dissociation (IRMPD) spectroscopy on IM-selected ions can provide additional details about the ions' molecular structure.^36,38^ In IRMPD, ions are dissociated in a mass spectrometer in a wavelength dependent manner.

**Fig. 1:**
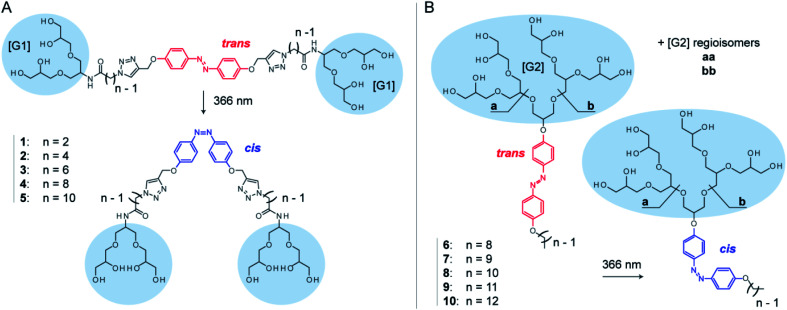
The gas-phase properties of two azobenzene-based detergent families are investigated: OGBAs and OGDs. (A) The structure of OGBAs **1–5** contains two [G1] trigylcerol head groups that are connected *via* variable linkers to an azobenzene core. (B) OGD batches **6–10** contain regioisomer mixtures of [G2] dendritic triglycerol (**aa**, **ab**, **bb**), which are connected to azobenzene and hydrocarbon chains of different lengths. Irradiation at 366 nm in solution causes *trans* to *cis* isomerization. Accompanied changes in structure and basicity of azobenzene enable the control over protein charge reduction by irradiation with light.

Here, we systematically investigate the molecular properties of azobenzene detergents and their utility for protein mass spectrometry. We focus on two detergent families: oligoglycerol bola-type detergents (OGBAs) and oligoglycerol detergents (OGDs, Fig. 1). We explore the utility of gas-phase techniques for quantifying their relative *cis*/*trans* ratios from solution and analyse how the molecular shape of these detergents is affected by (i) their isomeric state, (ii) general architecture, and (iii) structure of the hydrophobic backbone. By taking advantage of these findings we explore the utility of non-ionic azobenzene OGDs for the native MS analysis of membrane proteins.

## Results and discussion

### Azobenzene-based oligoglycerol detergents

OGBAs and OGDs consist of dendritic oligoglycerol, which is biocompatible and can be synthesized on large scales.^39–41^ The molecular architecture of both detergent families is modular: OGBAs **1–5** contain two first-generation [G1] triglycerol head groups which are connected *via* variable linkers to a central azobenzene core. OGD batches **6–10** consist of second-generation [G2] triglycerol regioisomer mixtures which are connected to azobenzene and hydrocarbon chains of different lengths (Fig. 1). Previous studies focused on the structure-based understanding of their self-assembly in solution and the potential of their aggregates to be used for the transport of hydrophobic guests.^23,24,27,42,43^ The self-assembly and macroscopic properties of OGBAs and OGDs depend on the length of the hydrophobic alkyl chains, the size of the head group, and the isomeric state of azobenzene. Changing the length of alkyl linkers in [G1] OGBAs, for example, leads to detergents which exhibit either photo-switchable water-solubility,^27^ photo-switchable aggregation behaviour,^24,27^ or a strong propensity to form photo-resistant H-aggregates.^27^ Studies on [G1]–[G3] OGDs revealed that a [G1] head group in combination with azobenzene and a hydrophobic alkyl spacer produces detergents that are poorly soluble in water.^23^ However, [G2] and [G3] OGDs are highly water-soluble and their aggregates can change their transport behaviour in response to UV light irradiation.^23,42^ Here, we take advantage of their modular architectures to study systematically the translation of their properties from solution to gas phase.

### Relative quantification of isomers ratios

**Fig. 2:**
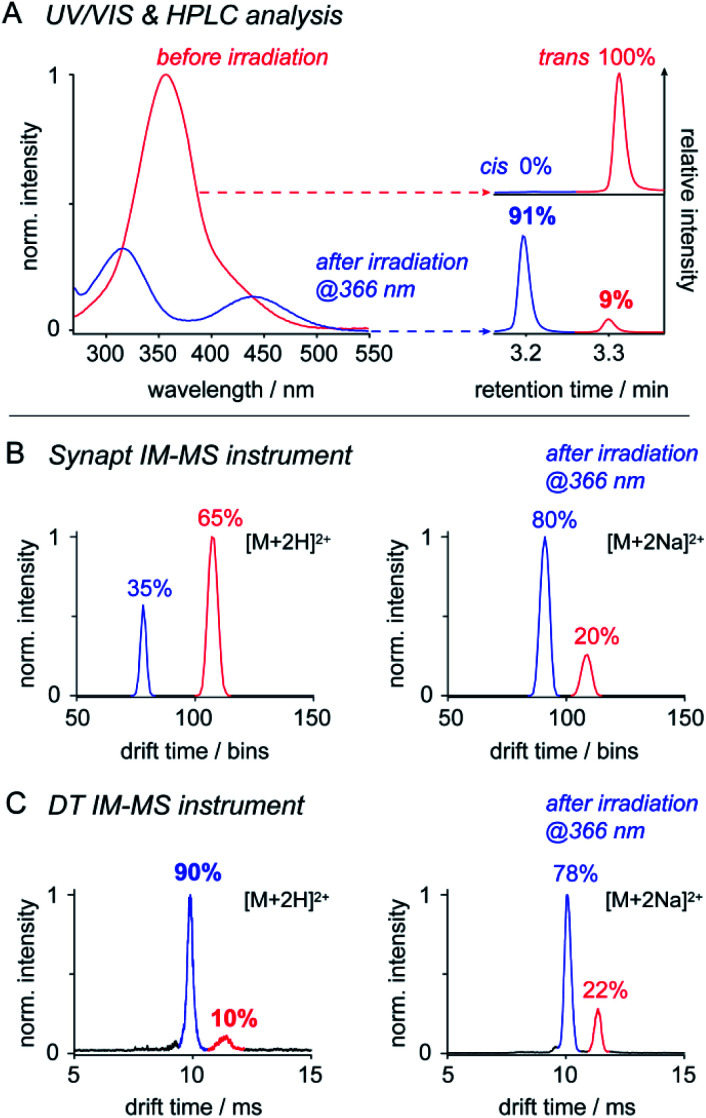
The utility of IM-MS for the relative quantification of *cis* and *trans***3**. (A) UV/VIS and HPLC analysis revealed a *cis*/*trans* ratio of 91/9 after irradiation of the sample solution at 366 nm. Further analysis with (B) a Synapt IM-MS instrument and (C) a DT IM-MS instrument revealed that relative intensities of doubly protonated *cis* and *trans* [M + 2H]^2+^ vary with the instrument type. Relative intensities of doubly sodiated ions [M + 2Na]^2+^ are instrument-independent. The *cis*/*trans* ratio obtained in solution was exclusively reproduced by (C) DT IM-MS analysis of [M + 2H]^2+^ ions. The ability to quantify *cis*/*trans* ratios by IM-MS depends on instrument conditions and ion properties.

IM-MS has been used to monitor the light-driven molecular shape transition and underlying isomerization kinetics of azobenzene derivatives.^24,44–47^ One of the remaining questions is, if IM-MS is capable providing data on the isomer ratios present in solution. To address this question, we first investigated the isomer ratios of OGBA **3** by using two different IM-MS instruments: (a) a commercially available Synapt G2 S HDMS travelling wave (TW) instrument and (b) a homebuilt linear drift tube (DT) instrument (Fig. S2, ESI†). UV/VIS and HPLC experiments showed that equilibration of an aqueous solution of **3** under irradiation at 366 nm leads to an isomer ratio of 91% *cis* and 9% *trans* (Fig. 2). Subsequent MS analysis revealed singly and doubly charged sodiated and/or protonated ions on both instruments (Fig. S3, ESI†). In line with previous results, baseline-separated arrival time distributions (ATDs) of *cis* and *trans* were obtained only from doubly charged ions (Fig. 2).^24^ In light of this result, we evaluated to what extent the relative *cis*/*trans* ratio of **3** in solution is reflected by the ATDs of double charged ions in the gas phase. For further experimental details see ESI†.

The relative intensity obtained from doubly protonated *cis***3** [M + 2H]^2+^ at the Synapt is about 60% lower compared to solution (Fig. 2). This indicates that *cis* to *trans* isomerization occurred between nanoelectrospray ionisation (nESI) and IM analysis, which is very likely a result of the non-continuous pressure profile of the Synapt instrument (Fig. S2, ESI†). In Synapts, ions traverse a high-vacuum region between the ion source and IM cell. Subsequent transfer and injection of the ions into the IM cell is accompanied by an increase in pressure of about four orders of magnitude. This causes collisional heating of the ions and is likely to induce *cis* to *trans* conversion prior the IM separation.^48,49^ In comparison, instrument pressure is continuously decreasing between the ion source and IM cell of typical DT instruments (Fig. S2, ESI†), thus leading to a softer ion transmission^50^ and a higher relative intensity of doubly protonated *cis* (Fig. 2). The isomer ratio obtained from DT analysis of doubly protonated *cis* matches with data obtained from solution (Fig. 2). In addition, our results suggest that the effect of collisional heating on the *cis*/*trans* ratio is supported by protonation of ions during nESI. Protonation of the *cis* N=N bond lowers the activation energy for the thermal *cis* to *trans* isomerization,^46^ which serves an additional explanation for the reduced relative intensity of doubly protonated *cis* under harsh instrument conditions (Fig. 2).

In contrast, relative intensities of doubly sodiated *cis***3** [M + 2Na]^2+^ were about 11% lower compared to solution, irrespective of the IM-MS instrument type (Fig. 2). In comparison to protons, sodium ions are more likely located at the crown ether-like head groups.^7^ The slightly reduced relative intensity of doubly sodiated *cis***3** might have two reasons: first, charge repulsion between sodiated head groups could lower the thermal stability of *cis*. Second, isomer ratios obtained from IM-MS may also be affected by the ionization efficiency of individual isomers and ion adducts.

In summary, only the DT IM-MS analysis of doubly protonated **3** revealed a *cis*/*trans* ratio that matches with solution data (Fig. 2, Table S1, ESI†). Further tests on OGDs **6**, **8**, and **10** led to comparable isomer ratios only in case of sodiated ions (Table S2, ESI†). This leads us to the conclusion that the ability to quantify *cis*/*trans* ratios from solution by IM-MS depends on both instrument conditions and properties of the investigated species. Our data underline that IM-MS enables the quantification of relative *cis*/*trans* ratios from solution. However, IM-MS may not be used as stand-alone technique. Reference data from solution are needed for the identification of IM-MS instrument conditions that deliver comparable results.

### Molecular shape transition

The molecular shape of azobenzene detergents influences their self-assembly in solution.^14,24,27^ IM-MS is sensitive to the overall shape of molecules and therefore a valuable tool to deduce changes in molecular shape of azobenzene-based compounds.^24,44,47^ To study how the shape of azobenzene detergents is affected by (i) the isomeric state, (ii) general molecular architecture, and (iii) structure of the hydrophobic backbone, we analysed the CCS values of *cis* and *trans***1–10**.

**Fig. 3:**
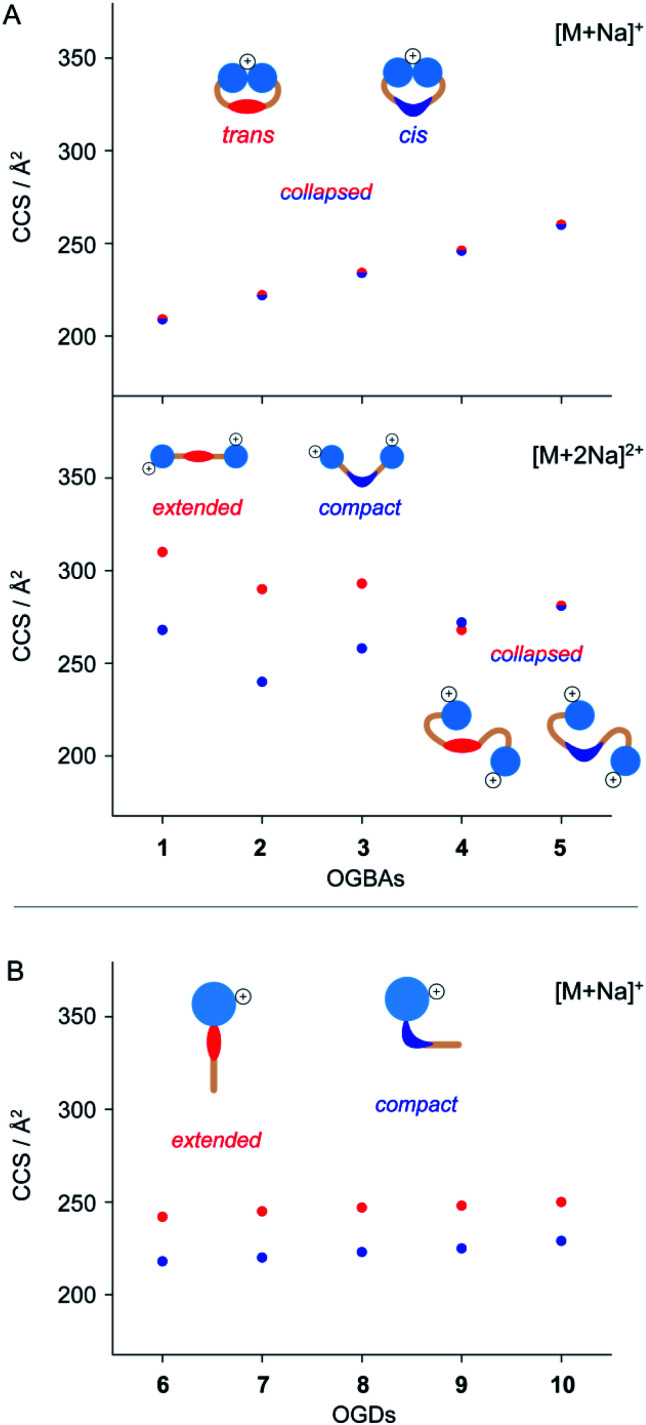
Investigating the impact of isomerization on the molecular shape of (A) singly and doubly charged OGBAs **1–5** and (B) singly charged OGDs **6–10**. IM separation of *cis* and *trans* azobenzene detergents depends on their general architecture, charge states, and relative size of the hydrophobic backbone. IM-MS analysis of doubly charged OGBAs and singly charged OGDs revealed that increasing the size of the hydrophobic backbone reduces the impact of isomeric state and charge state on the overall shape of azobenzene detergents. The experimental error of each CCS is less than 1% and smaller than the size of the symbols.

The CCS values obtained from singly charged *cis* and *trans* OGBAs **1–5** are similar, irrespective of the size of the hydrophobic backbone (Fig. 3). Charge interactions, either attractive or repulsive, are enhanced in the absence of solvent.^51^ For singly charged OGBAs, this causes very likely intramolecular interactions between the triglycerol head groups and forces the detergent into a more compact conformation. According to this scenario, distinct structural features of *cis* and *trans* are poorly reflected in the overall shape of the detergents (Fig. 3). In line with this explanation, two well-distinguishable CCS values are obtained for doubly charged ions of *cis* and *trans***1**, **2**, **3**, and **4** (Fig. 3). Here, charge-repulsive interactions prevent the head groups from intramolecular interactions and two distinguishable conformations are obtained (Fig. 3). Conversely, CCS values obtained from *cis* and *trans***5** are similar, irrespective of the charge state (Fig. 3). We conclude that increasing the size of the alkyl spacers reduces the impact of isomeric state and charge state on the overall shape of OGBAs (Fig. 3). This might have two reasons: first, elongating the alkyl spacers lowers the relative proportion of azobenzene in the overall detergent structure. Second, long alkyl spacers facilitate more conformational freedom.^27^

In comparison to OGBAs, only one head group is present in OGDs rendering a collapse of the gas-phase structure unlikely. As a consequence, clearly separated CCS values are obtained from singly charged *cis* and *trans* OGDs **6–10** (Fig. 3). This underlines that the ability to separate *cis* and *trans* by IM-MS depends not only on the charge state but also on the general architecture of the investigated species. Again, differences in CCS between *cis* and *trans* OGDs become smaller with increasing size of the hydrophobic backbone (Fig. S4, ESI†), thus indicating that this correlation can be extrapolated to other detergent classes. The length of alkyl spacers affects the polarity and shape of detergents.^27,52,53^ Our data underline that IM-MS allows us to analyse in addition how the overall shape of azobenzene detergents is affected by the interplay between (i) isomeric state, (ii) general molecular architecture, and (iii) size and flexibility of the hydrophobic backbone; all of which determine the aggregation behaviour of azobenzene detergents.^23,24,27,42^ In light of these results we anticipate that IM-MS could be used as a diagnostic tool that helps to tune the photo-switchable properties of supramolecular systems in the future.

### Switchable protonation sites

Very recently, it has been suggested that similar CCS values of singly protonated OGBAs result from the protonation of the *cis* N=N bond during nESI, which causes a quantitative reconversion to *trans* prior to IM analysis.^46^ Intramolecular vibrations of azobenzene depend on its isomeric state, particularly in the mid-IR range.^54^ This makes gas-phase IR spectroscopy a valuable tool for evaluating if the isomeric state of singly protonated OGBAs retains under the experimental conditions employed.^55^ Here, we exemplarily investigated the gas-phase structure of singly protonated OGBA **1** after IM separation by gas-phase IRMPD spectroscopy. The IM analysis of singly protonated **1** [M + H]^+^ revealed a minor shift in the ATD to lower drift times after irradiation of the sample solution at 366 nm. The experimental CCS values indicate that *trans***1** (208 Å^2^) adopts a slightly more compact conformation upon irradiation at 366 nm (206 Å^2^).

Similar theoretical CCS values were obtained from simulated conformers of *trans***1** (208 ± 2) Å^2^ and *cis***1** (206 ± 2) Å^2^ in which both head groups stick together (Fig. S5, ESI†). However, the differences in theoretical CCS values are similar within their errors rendering a clear explanation of the experimental result by molecular dynamics simulations difficult.

**Fig. 4:**
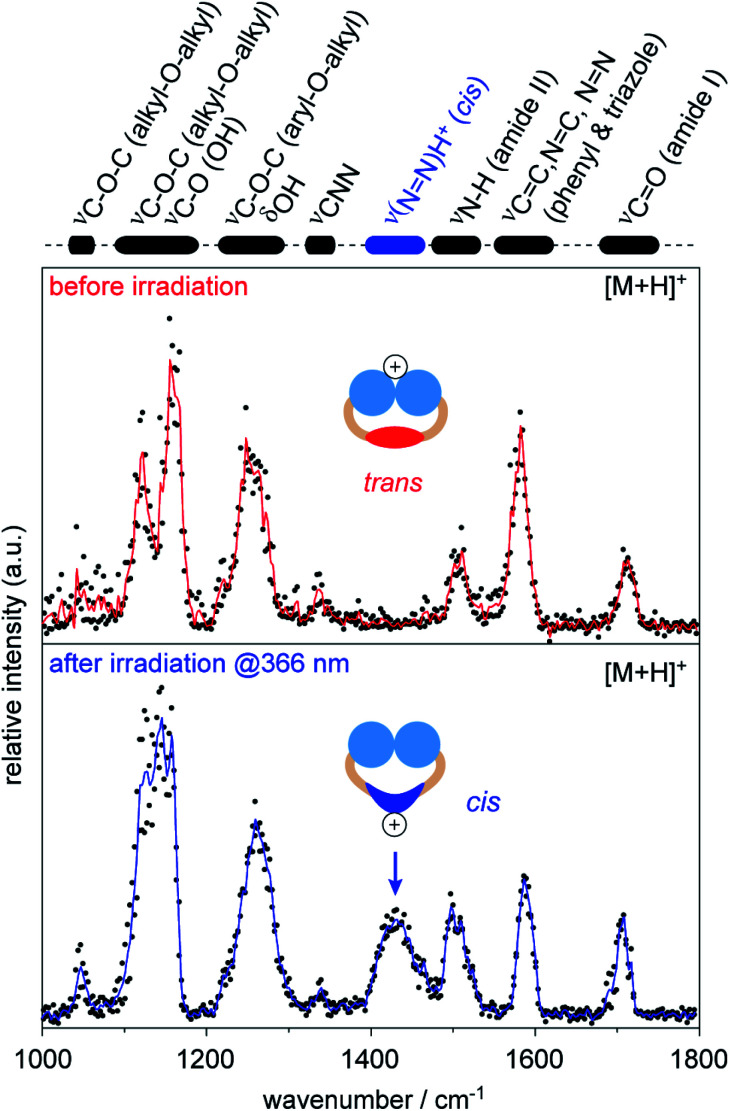
Gas-phase IRMPD spectra of singly protonated **1** [M + H]^+^ obtained before and after irradiation of the sample solution at 366 nm. Wavenumbers obtained from amide bands (I + II) and aromatic backbone (phenyl & triazole) are representative for unprotonated groups and indicate that protonation in *trans***1** mainly occurred at the head groups. After irradiation at 366 nm, an additional band appeared around 1430 cm^−1^, which indicates that protonation mainly occurred at the *cis* N=N bond. The protonation sides of azobenzene detergents can be changed by irradiation with light.

Therefore, we investigated singly protonated ions of **1** by IRMPD spectroscopy after they have passed the IM cell (Fig. S6, ESI†). The spectrum obtained from singly protonated *trans***1** shows multiple features: amide I band (∼1700 cm^−1^), aromatic backbone (∼1600 cm^−1^), amide II band (∼1500 cm^−1^), CNN band (1340 cm^−1^), and overlapping bands from the head groups (<1300 cm^−1^, Fig. 4).^54,56,57^ The structure of *trans***1** offers multiple protonation sites, including the head groups, carbonyl groups, triazoles, and the N=N bond. The obtained IR bands match with those of unprotonated carbonyl and triazole groups and provide no evidence that protonation of the *trans* N=N group occurred.^54,56,57^ This leads to the conclusion that the triglycerol head groups are the main protonation sites of *trans***1** during nESI. Surprisingly, the ether and hydroxyl groups of the triglycerol head groups are associated with lower gas-phase basicity values than other building blocks (Table S3, ESI†). This indicates that the preferred protonation site of a molecule depends not only on the gas-phase basicity but also on the number and accessibility of its functional groups.^58^

The IRMPD spectrum obtained after irradiation of the sample solution at 366 nm represents an average of a singly protonated *cis*/*trans* mixture, because the light-driven isomerization in solution is not quantitative and protonated isomers of **1** are not separable by IM (Fig. S5, ESI†). However, an additional IR band appeared at 1430 cm^−1^ after irradiation at 366 nm (Fig. 4). The obtained wavenumber is lower than values reported for the protonated N=N bond of unsubstituted *cis* azobenzene (1478–1515 cm^−1^).^54^ This is expected since it is known that the electron-donating alkoxy groups of azobenzene lower the double-bond character of the protonated N=N bond (Fig. S7, ESI†).^54,56^ The appearance of the new band at 1430 cm^−1^ together with changes in relative intensities of head group vibrations (<1200 cm^−1^) indicate that the average protonation site of **1** changed to the N=N bond after irradiation at 366 nm. Taken together, our data underline that protonated *cis***1** retains its double bond configuration during nESI and IM analysis under the experimental conditions employed.

### Membrane protein charge reduction

The change in protonation sites of OGBA **1** can be understood as a consequence of the light-driven change in N=N bond basicity. The basicity of detergents has recently been identified as a key parameter for controlling charge reduction in membrane protein mass spectrometry.^7,59^ This prompted an important question. Can azobenzene detergents be used for controlling protein charge reduction by light?

**Fig. 5:**
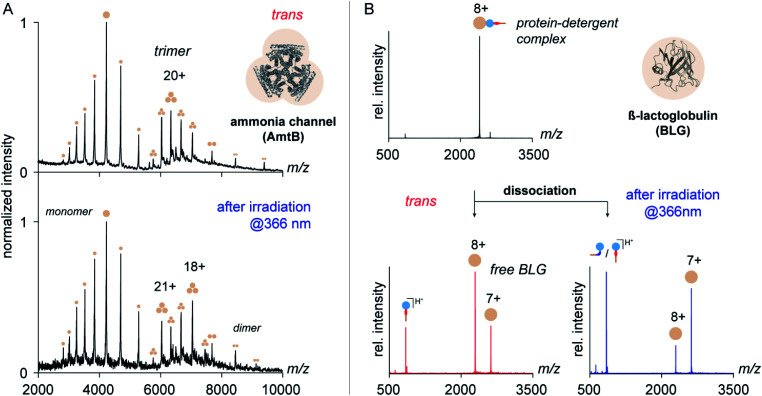
The utility of azobenzene OGDs for the native MS analysis of membrane proteins. (A) Native MS analysis of AmtB following detergent exchange from DDM to *trans* OGD **6** revealed that the native trimeric state of AmtB is preserved. After irradiation at 366 nm, lower charge states appeared for the AmtB trimer. (B) Gas-phase dissociation of PDCs (*z* = 8+) formed by the amphiphilic protein BLG and OGD **6** confirms the impact of the *trans* to *cis* isomerization on protein charge reduction. Partial charge reduction of BLG was obtained upon dissociation of PDCs formed with *trans***6** (left). Charge reduction was substantially enhanced after irradiation of the sample solution at 366 nm (right). OGD **6** presents the first example of an azobenzene detergent that enables the native MS analysis of a membrane protein complex and whose charge-reducing properties can be changed by irradiation with light.

To address this question, we purified the trimeric ammonia channel (AmtB) from cell membranes of *E. coli* using *n*-dodecyl-β-d-maltoside (DDM) and exchanged the detergent environment from DDM to *trans* OGD **6** using size-exclusion chromatography. The so-obtained membrane protein solution was stable over multiple freeze–thaw cycles, before and after irradiation at 366 nm, which confirms the utility of *trans***6** and its *cis*/*trans* mixture to solubilize and stabilize membrane proteins. To investigate if the native quaternary structure of AmtB was preserved upon detergent exchange, we analysed the samples by native MS. The mass spectra obtained before and after irradiation at 366 nm revealed AmtB in its native trimeric state together with gas-phase dissociation products, such as monomers and dimers.^8^ This shows that *trans***6** and its *cis*/*trans* mixture cannot only solubilize but also preserve the native oligomeric state of AmtB. The most abundant charge state of AmtB obtained from the MS analysis with *trans***6** (21+) is similar to that obtained from DDM (22+), which is classified as a non-charge-reducing detergent (Fig. 5).^6^ Upon irradiation at 366 nm, a bimodal charge state distribution appeared and the most abundant charge state was shifted to higher *m*/*z* values (18+, Fig. 5).

While the shift in the most abundant charge state indicates that *cis***6** is more charge-reducing than *trans***6**, the bimodal charge state distribution also indicates that AmtB partially unfolded.^60^ To investigate if the protein unfolded in solution, we analysed the secondary structure of AmtB by circular dichroism (CD) spectroscopy. We obtained a similar alpha-helical content in DDM and **6**, before and after irradiation at 366 nm (Fig. S8, ESI†). We conclude that *trans***6** as well as its *cis*/*trans* mixture can preserve the native fold of AmtB in solution. The bimodal charge state distribution obtained upon irradiation at 366 nm reflects therefore likely partial protein unfolding during nESI.^60^

Detergents are in many ways important for the native MS analysis of membrane proteins as they can protect the native structure in solution and during the nESI process.^6,8,59^ The detergent concentration used for solubilising membrane proteins is often adjusted to a multiple of the detergents' critical aggregation concentration (cac).^8^ Attempts to solubilise AmtB with concentrations below the cac of *trans***6** led to protein precipitation, thus underlining that at least concentrations close to the cac are needed to stabilize AmtB in aqueous buffer. The different charge state distributions obtained for AmtB, before and after irradiation at 366 nm, motivated us to further investigate the properties of the detergent micelles in solution. Interestingly, similar cac values were obtained for **6**, before and after irradiation at 366 nm (Fig. S9, ESI†). The diffusion coefficients of the aggregates were similar and in the same order of magnitude as values reported from other micelle-forming [G2] OGDs (Fig. S9, ESI†).^41^ This indicates that *trans***6** as well as its *cis*/*trans* mixture are capable of forming micelles. While investigating the molecular properties of our azobenzene detergents we found that the *trans* to *cis* isomerization is affecting the geometry and polarity of the hydrophobic tail (Fig. 3 and 4). Micelles formed by the *cis*/*trans* mixtures are more heterogeneous than those solely formed by *trans* azobenzene detergents.^23^ It is very likely that the bent and more polar *cis* form disturbs the packing of the micelles.^23^ In this way, *cis***6** could lower the efficiency of the micelle in shielding the hydrophobic protein surface and facilitate, for example, partial protein unfolding during nESI.

To gain more insights into the charge-reducing properties of **6**, we extended our studies on the soluble protein β-lactoglobulin (BLG). This protein contains an amphiphilic pocket that is capable of forming non-covalent complexes with detergents.^7^ Unlike membrane proteins, BLG is not encapsulated in micelles and serves as an ideal model system to study the impact of individual functional groups of detergents on the ability to reduce the charge of protein ions in the gas phase.^7^ For this purpose, OGDs and BLG were mixed in solution and analysed by nESI-MS using a modified Ultima high-mass quadrupole time-of-flight mass spectrometer (for further details see ESI†). Subsequently, the gas-phase dissociation behaviour of protein–detergent complexes (PDCs) formed by **6** and BLG was analysed by tandem mass spectrometry (MS/MS). Closer analysis of the dissociation products revealed how the isomerization of azobenzene mediates the ability of **6** to remove protons from protein ions within the vacuum of a mass spectrometer.

Mass spectra obtained from equimolar mixtures of OGD **6** and BLG show a narrow protein charge state distribution with charge states ranging from 6+ to 10+ (Fig. S10, ESI†). To take into account in our analysis that the dissociation behaviour of non-covalent protein complexes varies significantly with the protein charge state,^61^ MS/MS experiments were focused on the most abundant protein charge state 8+ (Fig. 5). Upon dissociation of fully protonated PDCs, three new ion populations appeared. They could be assigned as protonated detergent ions and BLG of two different charge states (8+ and 7+, Fig. 5). Before irradiation of the sample solution, the most intense protein charge state belongs to non-charge reduced BLG (8+, Fig. 5). After irradiation, the most intense protein charge state belongs to charge-reduced BLG (7+, Fig. 5). This confirms that the increase in N=N bond basicity of azobenzene during *trans* to *cis* isomerization affects the detergent's propensity to pick up a charge from the protein ion during PDC dissociation.

To analyse underlying changes in basicity of azobenzene, MS/MS experiments were repeated with OGDs in which azobenzene was substituted for linker groups with different gas-phase basicity values, such as ether (794 kJ mol^−1^), amide (820 kJ mol^−1^), and triazole (925 kJ mol^−1^, Fig. S11,† Table S3, ESI†).^7,62,63^ The fraction of charge-reduced BLG (%) obtained upon PDC dissociation was plotted against the gas-phase basicity values of the linker groups and the data were fitted to a linear function. An extrapolation to the gas-phase basicity values of **6** indicates that its average gas-phase basicity went up by 60 kJ mol^−1^ after irradiation of the sample solution at 366 nm (Fig. S12†). This implies that optimizing the isomerization yields and gas-phase basicity values of *cis* can maximize protein charge reduction. To the best of our knowledge, OGD **6** presents the first example of an azobenzene detergent that enables the native MS analysis of a membrane protein complex and whose charge-reducing properties can be changed by irradiation with light.

## Conclusions

In summary, we have investigated light-induced property changes of azobenzene detergents with different gas-phase techniques. The ability to quantify isomer ratios of azobenzene detergents in solution by IM-MS depends on instrument conditions and ion properties. This demonstrates the importance of reference data from solution for identifying IM-MS instrument conditions that deliver comparable results. Moreover, the ability to separate *cis* and *trans* isomers of azobenzene detergents by IM-MS depends on their molecular architecture, charge state, and the structure of the hydrophobic backbone. Generally, increasing the size and flexibility of the hydrophobic backbone reduces the impact of the isomeric state on the overall shape of detergents. We expect that our findings will have a sustained effect on (i) the application of IM-MS for quantifying isomer ratios of azobenzene compounds in solution and (ii) the identification of molecular design rules that will lead to predictable property changes in azobenzene detergents. Both aspects are of great importance for tuning the properties of photo-switchable supramolecular systems. In addition, our data show that light-driven changes in basicity influence the protonation sites of azobenzene detergents. This led us to the first azobenzene detergent that enables a non-denaturing structural analysis of membrane proteins and whose charge-reducing properties for protein mass spectrometry can be tuned by irradiation with light. Azobenzene offers a new design strategy for gaining control over charge-reducing properties of detergents. We expect that optimizing isomerization yields and increasing the basicity of *cis* are key parameters for unlocking their full potential for native MS of membrane proteins. In light of these results, we anticipate fine-tuning the protein-compatibility of azobenzene detergents in solution and gas phase in the future.

## Author contributions

L. H. U and R. H. designed the detergents; L. H. U., M. S., and C. M. synthesized the detergents; L. H. U., M. S., Y. B. M., S. W., I. L., C. V. R., and K. P. conceived the experiments; L. H. U., M. S., W. H., Y. B. M., S. W., I. L., and K. F. performed the experiments; all authors co-wrote the paper.

## Conflicts of interest

There are no conflicts to declare.

## Supplementary Material

SC-011-D0SC01022G-s001
