# Supplementary material for: A new azobenzene-based design strategy for detergents in membrane protein research
Source: Chem Sci. 2020 Mar 13;11(13):3538–46. doi: 10.1039/d0sc01022g (PMC8152689; doi:10.1039/d0sc01022g)
Supplement: SC-011-D0SC01022G-s001 [file SC-011-D0SC01022G-s001.pdf]

Electronic Supplementary Material (ESI) for Chemical Science.
This journal is © The Royal Society of Chemistry 2020

Supporting Information

for

A New Azobenzene-Based Design Strategy for Detergents in Membrane Protein Research

Leonhard H. Urner,[a,b,c*] Maiko Schulze,[a] Yasmine B. Maier,[a] Waldemar Hoffmann,[a,b] Stephan Warnke,[b] Idlir Liko,[c] Christian Manz,[a] Kristin Folmert,[a] Carol V. Robinson,[c] Rainer Haag,[a] Kevin Pagel[a,b*]

[a]*Freie Universität Berlin, Institute of Chemistry and Biochemistry, Takustraße 3, 14195 Berlin, Germany.*

[b]*Fritz Haber Institute of the Max Planck Society, Department of Molecular Physics, Faradayweg 4-6, 14195 Berlin, Germany.*

[c]*Physical and Theoretical Chemistry Laboratory, University of Oxford, South Parks Road, OX13QZ Oxford, United Kingdom*

Table of Contents

Synthesis and Characterization 1
Quantification of Isomers in Solution 6
Ion Mobility-Mass Spectrometry 7
Gas-Phase Infrared Spectroscopy 11
Membrane Protein Solubilisation 12
Protein Mass Spectrometry 14
Literature 17

Synthesis and Characterization

General Remarks to Synthesis. OGBAs and OGDs were synthesized by means of previously published procedures.[1-3] Synthesis protocols that led to the obtainment of OGBAs **1 – 5** have been published before.[2, 3] The general synthesis route that was used to obtain OGDs has also been published before.[1] In particular, the detergent batches **6 – 10** are new and related synthesis protocols have not been reported before.

Chemicals were purchased from Sigma-Aldrich (Germany), Acros Organics (Germany), Alfa Aesar (Germany), Fluka (Germany), Fisher Scientific (Germany), Merck (Germany), TCI (Germany). Chemicals were used as supplied. Ethyl acetate (EtOAc) and *n*-hexane were distilled before they were used. Other solvents, such as methanol (MeOH), dimethylformamide (DMF), dichloromethane (DCM), and acetonitrile (ACN), were used as supplied. Dry solvents were purchased in bottles sealed with a septum or tapped from a solvent purification system (MS-SPS-800) that was purchased from M. Braun (Germany). Deionized water (H_2O) used for synthesis was provided by a deionization system installed in the Freie Universität's Institute of Chemistry and Biochemistry. Argon was purchased from Linde (Germany). For working under dry and oxygen-free reaction conditions, chemicals and solvents were handled under argon atmosphere. To support dry conditions the glassware was evacuated, heated up to 300 °C with a heat gun, and filled with argon. Dendritic compounds, such as mesylated and acetal-protected [G2] dendron ([pG2]-OMes), and 4,4´-dihydroxyazobenzene were synthesized in analogy to previously published procedures.[1, 3-5]

Monitoring of reactions and purification procedures was achieved by normal phase (NP) thin-layer chromatography (TLC) analysis. NP TLC plates (DC-Fertigfolien ALUGRAM® Xtra SIL G/UV254) based on silica (SiO_2) were purchased from Macherey-Nagel (Germany). Silica gel (60 M) for preparative normal phase column chromatography was purchased from Macherey-Nagel. For NP TLC analysis and manual NP column purification mixtures of organic solvents (*v:v*) were prepared. If necessary, MeOH was added in percent per volume to the prepared mixtures (*v*:*v* + *v*%). TLC plates were either analyzed under UV irradiation (254 nm) using a lamp from CAMAG (Germany) or by staining the TLC plates with cerium reagent (940 mL H_2O, 60 mL H_2SO_4, 25 g molybdic acid, 10 g cerium(IV) sulfate). For the staining process, the TLC plates were fully submerged into the cerium reagent, excess of staining reagent was wiped off with cellulose, and the plate was heated up to 300 °C with a heat gun until staining was completed.

For high-pressure liquid chromatography (HPLC) purifications a setup from Knauer was used, which consisted of a Smartline Manager 5000 (+ interface-module), two Smartline Pumps 1000, a 6-port-3-channel-injection valve, a sample loop (10 mL), UV Detektor 2500 und a high pressure gradient mixer. Spectra were recorded with a x-y-plotter (Knauer). A reversed-phase (RP) Luna™ C18(2) (10 µm, 250 x 21.20 mm, Phenomenex®) column was used as stationary phase, which was further equipped with a Security Guard™ PREP Cartridge Holder Kit (21.20 mm, ID, Phenomenex®). The setup was constructed by Dr. Carlo Fasting. Components of the mobile phase, such as H_2O and ACN, were supplemented with 0.1% trifluoroacetic acid and degassed prior usage. The flow rate was adjusted to 20 mL/min and the detection wavelength was 360 nm.

Mass spectra were acquired on an Agilent 6210 ESI-TOF (ESI-ToF) from Agilent Technologies (Santa Clara, CA, USA). The solvent flow rate was adjusted to 4 µL/min and the spray voltage was set to 4 kV. Drying gas flow rate was set to 15 psi (1 bar). All other parameters were adjusted for a maximum abundance of the relative $[M+H]^+$. The instrument was operated by the Core Facility BioSupraMol of the Freie Universität Berlin. ^1H NMR, ^{13}C NMR and DEPT135 spectra were acquired using the following NMR instruments: Bruker DPX400 (^1H NMR: 400 MHz, ^{13}C NMR: 100 MHz), Jeol ECX400 (^1H NMR: 400 MHz, ^{13}C NMR: 100 MHz), Jeol ECP 500 (^1H NMR: 500 MHz, ^{13}C NMR: 125 MHz), Bruker AVANCEIII500 (^1H NMR: 500 MHz, ^{13}C NMR: 125 MHz) or Bruker AVANCEIII700 (^1H NMR: 700 MHz, ^{13}C NMR: 175 MHz). All instruments were operated by the Core Facility BioSupraMol of the Freie Universität Berlin. NMR data were processed with MestReNova (*v*6.0.2-5475).

General Procedure for the Synthesis of C_n-OMes (n = 8 – 12)

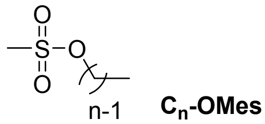

The desired alcohol (5 g) was dissolved in toluene (150 mL). Triethylamine was added (2 eq.) and the reaction flask was cooled with an mixture of water and ice (*w*:*w*, 1:1). Methanesulfonyl chloride (1.2 eq.) was added and the mixture was allowed to warm up to room temperature over a time period of 16 hours. Massive precipitation of triethylamine hydrochloride sometimes interrupted the stirring process. In this case, more toluene was added and the precipitate was suspended manually using a spatula. After 16 hours, the precipitate was filtered off with a filter paper and solvent was removed under reduced pressure. Column chromatography (SiO_2, DCM) gave the desired product as colorless oil (n = 8, 91%; n = 9, 97%; n = 10, 98%; n = 11, 99%; n = 12, 69%).

C_8-OMes (n = 8): ^{1}H NMR ($CDCl_3$, 400 MHz) δ 4.21 (t, 2H), 3.00 (s, 3H), 1.74 (m, 2H), 1.43 - 1.23 (m, 10H), 0.88 (t, 3H). ^{13}C NMR ($CDCl_3$, 100 MHz) δ 70.3, 37.4, 31.8, 29.2, 29.1, 25.5, 22.7, 14.1. Exact mass calculated for $C_9H_{20}O_3S_1Na_1^+$ ($[M+Na]^+$): 231.1025; mass found: 231.1031.

C_9-OMes (n = 9): ^{1}H NMR ($CDCl_3$, 400 MHz) δ 4.21 (t, 2H), 2.99 (s, 3H), 1.74 (m, 2H), 1.40 - 1.20 (m, 12H), 0.87 (t, 3H). ^{13}C NMR ($CDCl_3$, 100 MHz) δ 70.3, 37.4, 31.9, 29.4, 29.2, 29.1, 25.5, 22.7, 14.2. Exact mass calculated for $C_{10}H_{22}O_3S_1Na_1^+$ ($[M+Na]^+$): 245.1182; mass found: 245.1213.

C_{10}-OMes (n = 10): ^{1}H NMR ($CDCl_3$, 400 MHz) δ 4.22 (t, 2H), 3.00 (s, 3H), 1.74 (m, 2H), 1.43 - 1.22 (m, 14H), 0.88 (t, 3H). ^{13}C NMR ($CDCl_3$, 100 MHz) δ 70.4, 37.5, 32.0, 29.6, 29.4, 29.3, 29.2, 25.6, 22.8, 14.2. Exact mass calculated for $C_{11}H_{24}O_3S_1Na_1^+$ ($[M+Na]^+$): 259.1338; mass found: 259.1385.

C_{11}-OMes (n = 11): ^{1}H NMR ($CDCl_3$, 400 MHz) δ 4.22 (t, 2H), 3.00 (s, 3H), 1.74 (m, 2H), 1.44 - 1.22 (m, 16H), 0.87 (t, 3H). ^{13}C NMR ($CDCl_3$, 100 MHz) δ 70.3, 37.4, 32.0, 29.6, 29.5, 29.4, 29.2, 29.1, 25.5, 22.8, 14.2. Exact mass calculated for $C_{12}H_{26}O_3S_1Na_1^+$ ($[M+Na]^+$): 273.1495; found: 273.1505.

C_{12}-OMes (n = 12): ^{1}H NMR ($CDCl_3$, 400 MHz) δ 4.21 (t, 2H), 2.99 (s, 3H), 1.74 (m, 2H), 1.42 - 1.22 (m, 18H), 0.87 (t, 3H). ^{13}C NMR ($CDCl_3$, 100 MHz) δ 70.3, 37.5, 32.0, 29.7, 29.6, 29.5, 29.3, 29.2, 25.6, 22.8, 14.2. Exact mass calculated for $C_{13}H_{28}O_3S_1Na_1^+$ ($[M+Na]^+$): 287.1651; found: 287.1659.

General Procedure for the Synthesis of HOazoC_n (n = 8 – 12)

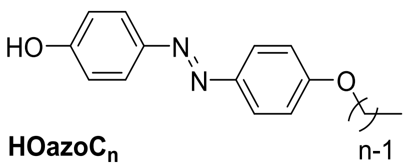

4,4´-Dihydroxyazobenzene[3] (500 mg, 2.33 mmol) was dissolved in dry DMF (25 mL). Potassium carbonate (2 eq.) was added and the suspension was heated up to 100 °C. A solution of the desired C_n-OMes (0.95 eq., 50 mM in DMF) was added drop wise over a time period of one hour. The mixture was stirred at 100 °C for 18 hours and then allowed to cool down to room temperature. The solvent was removed under reduced pressure and the remaining solid material was suspended in a mixture of H_2O (100 mL) and concentrated hydrochloric acid (1 mL, 37%). The aqueous layer was extracted with DCM. Organic layers were combined and dried over magnesium sulfate, and magnesium sulfate was filtered off using a filter paper. The raw product was immobilized on silica gel by adding SiO_2 (25 – 50 g) to the filtrate and removing the solvent under reduced pressure. Subsequent column chromatography (SiO_2, DCM) gave the desired product (n = 8, 33%; n = 9, 55%; n = 10, 25%; n = 11, 33%; n = 12, 27%).

HOazoC$_8$ (n = 8): ^{1}H NMR ($CDCl_3$, 400 MHz) δ 7.90 - 7.79 (m, 4H), 7.02 - 6.88 (m, 4H), 6.05 - 5.55 (m, 1H), 4.02 (t, 2H), 1.81 (m, 2H), 1.53 - 1.24 (m, 10H), 0.90 (t, 3H). ^{13}C NMR ($CDCl_3$, 100 MHz) δ 161.5, 158.0, 147.2, 146.8, 124.7, 124.5, 116.0, 114.9, 68.5, 32.0, 29.5, 29.4, 29.3, 26.2, 22.8, 14.3. Exact mass calculated for $C_{20}H_{27}N_2O_2^+$ ([M+H]$^+$): 327.2067; found: 327.2073.

HOazoC$_9$ (n = 9): ^{1}H NMR ($CDCl_3$, 400 MHz) δ 7.88 - 7.82 (m, 4H), 7.00 - 6.92 (m, 4H), 5.88 - 5.30 (m, 1H), 4.03 (t, 2H), 1.81 (m, 2H), 1.55 - 1.18 (m, 12H), 0.89 (t, 3H). ^{13}C NMR ($CDCl_3$, 100 MHz) δ 161.4, 158.0, 147.1, 146.7, 124.7, 124.5, 115.9, 114.8, 68.5, 32.0, 29.6, 29.5, 29.4, 29.3, 26.1, 22.8, 14.2. Exact mass calculated for $C_{20}H_{27}N_2O_2^+$ ([M+H]$^+$): 341.2224; found: 341.2265.

HOazoC$_{10}$ (n = 10): ^{1}H NMR ($CDCl_3$, 400 MHz) δ 7.89 - 7.80 (m, 4H), 7.01 - 6.90 (m, 4H), 5.60 - 5.40 (m, 1H), 4.03 (t, 2H), 1.81 (m, 2H), 1.52 - 1.23 (m, 14H), 0.89 (t, 3H). ^{13}C NMR ($CDCl_3$, 100 MHz) δ 161.5, 158.0, 147.2, 146.8, 124.8, 124.5, 116.0, 114.9, 68.5, 32.0, 29.7, 29.5, 29.4, 29.2, 22.8, 14.3. Exact mass calculated for $C_{22}H_{31}N_2O_2^+$ ([M+H]$^+$): 355.2380; found: 355.2400.

HOazoC$_{11}$ (n = 11): ^{1}H NMR (Aceton-d$_6$, 400 MHz) δ 7.95 - 7.80 (m, 4H), 7.05 - 6.90 (m, 4H), 5.65 - 5.25 (m, 1H), 4.03 (t, 2H), 1.81 (m, 2H), 1.53 - 1.22 (m, 16H), 0.88 (t, 3H). ^{13}C NMR (Aceton-d$_6$, 100 MHz) δ 162.1, 160.9, 147.6, 147.1, 125.3, 124.9, 116.6, 115.6, 68.9, 32.6, 30.3, 30.1, 29.9, 26.7, 23.3, 14.4. Exact mass calculated for $C_{23}H_{33}N_2O_2^+$ ([M+H]$^+$): 369.2537; found: 369.2546.

HOazoC$_{12}$ (n = 12): ^{1}H NMR ($CDCl_3$, 400 MHz) δ 7.93 - 7.81 (m, 4H), 7.02 - 6.93 (m, 4H), 5.75 - 5.30 (m, 1H), 4.03 (t, 2H), 1.81 (m, 2H), 1.52 - 1.22 (m, 18H), 0.88 (t, 3H). ^{13}C NMR ($CDCl_3$, 100 MHz) δ 161.5, 158.1, 147.2, 146.7, 124.8, 124.6, 116.0, 114.9, 68.5, 32.1, 29.8, 29.7, 29.5, 29.4, 26.2, 22.8, 14.3. Exact mass calculated for $C_{24}H_{35}N_2O_2^+$ ([M+H]$^+$): 383.2693; found: 383.2635.

General Procedure for the Synthesis of [pG2]azoCn (n = 8 – 12)

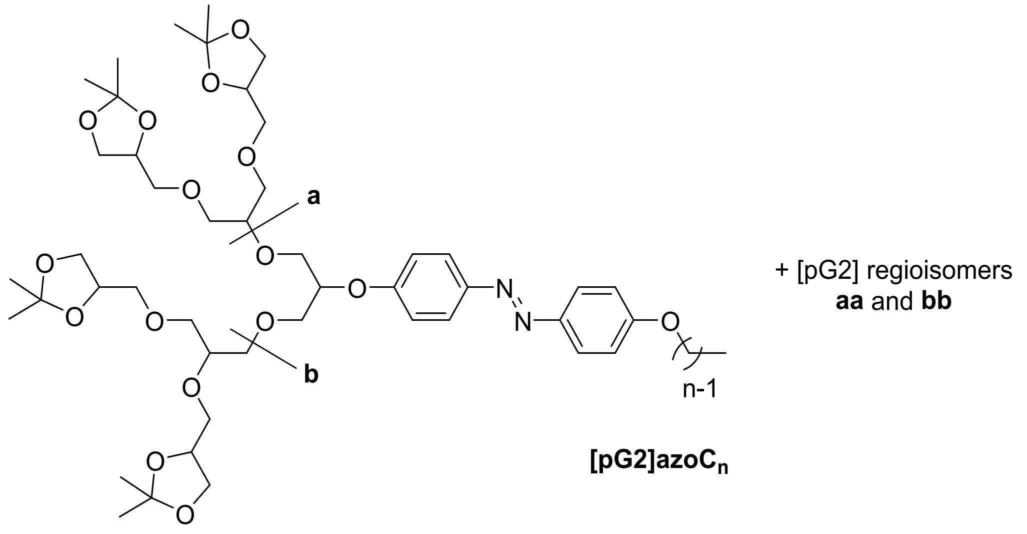

[pG2]azoC$_n$

The desired HOazoC$_n$ derivative (200 – 300 mg) was dissolved in dry DMF (5 mL) and potassium carbonate (1 eq.) was added. The mixture was heated up to 100 °C and a solution of a [pG2]-OMes[1, 4, 5] regioisomer mixture (1.1 eq., in 5 mL dry DMF) was added. The mixture was stirred at 100 °C for 18 hours and then allowed to cool down to room temperature. Solvent was removed under reduced pressure. The remaining material was suspended in H_2O (20 mL) and extracted with DCM. The organic layers were dried over magnesium sulphate. Subsequent column chromatography (SiO_2, DCM/EtOAc + MeOH, 8:2 + 1% → 20%) gave the desired product as orange-coloured oil (n = 8, 22%; n = 9, 49%; n = 10, 10%; n = 11, 23%; n = 12, 43%).

[pG2]azoC$_8$ (n = 8): ^{1}H NMR (MeOD-d$_4$, 500 MHz) δ 7.87 - 7.84 (m, 4H), 7.17 - 7.13 (m, 2H), 7.05 - 7.02 (m, 2H), 4.73 - 4.66 (m, 1H), 4.27 - 3.44 (m, 36H), 1.84 - 1.77 (m, 2H), 1.53 - 1.46 (m, 2H), 1.42 - 1.29 (m, 32H), 0.92 (t, 3H). ^{13}C NMR (MeOD-d$_4$, 125 MHz) δ 162.9, 148.5, 148.1, 125.4, 125.3, 117.4, 115.8, 110.5, 80.1, 76.1, 73.5, 72.5, 70.7, 69.4, 67.6, 33.0, 30.5, 30.4, 27.2, 25.7, 23.7, 14.5. Exact mass calculated for $C_{53}H_{84}N_2O_{16}Na_1^+$ ([M+Na]$^+$); 1027.5713; found: 1027.5828.

[pG2]azoC$_9$ (n = 9): ^{1}H NMR (MeOD-d$_4$, 500 MHz) δ 7.93 - 7.89 (m, 4H), 7.22 - 7.18 (m, 2H), 7.09 - 7.05 (m, 2H), 4.77 - 4.69 (m, 1H), 4.32 - 3.48 (m, 36H), 1.86 - 1.80 (m, 2H), 1.56 - 1.48 (m, 2H), 1.46 - 1.32 (m, 34H), 0.96 (t, 3H). ^{13}C NMR (MeOD-d$_4$, 125 MHz) δ 162.8, 161.9, 148.4, 148.1, 125.4, 125.3, 117.4, 115.8, 110.4, 80.1, 76.0, 73.5, 72.5, 70.7, 69.4, 67.6, 33.0, 30.7, 30.5, 30.4, 27.2, 25.8, 23.7, 14.6. Exact mass calculated for $C_{54}H_{86}N_2O_{16}Na_1^+$ ([M+Na]$^+$): 1041.5870; found: 1041.5881.

[pG2]azoC$_{10}$ (n = 10): ^{1}H NMR (MeOD-d$_4$, 500 MHz) δ 7.92 - 7.87 (m, 4H), 7.22 - 7.18 (m, 2H), 7.10 - 7.06 (m, 2H), 4.78 - 4.72 (m, 1H), 4.33 - 3.49 (m, 36H), 1.89 - 1.82 (m, 2H), 1.58 - 1.51 (m, 2H), 1.47 - 1.31 (m, 36H), 0.95 (t, 3H). ^{13}C NMR (MeOD-d$_4$, 125 MHz) δ 162.9, 148.5, 148.1, 125.4, 125.3, 117.4, 115.8, 110.5, 80.2, 76.1, 73.5, 72.2, 70.7, 69.4, 67.6, 33.1, 30.7, 30.5, 27.1, 25.7, 14.5. Exact mass calculated for $C_{55}H_{88}N_2O_{16}Na_1^+$ ([M+Na]$^+$): 1055.6026; found: 1055.6060.

[pG2]azoC$_{11}$ (n = 11): ^{1}H NMR (MeOD-d$_4$, 500 MHz) δ 7.87 - 7.83 (m, 4H), 7.15 (d, 2H), 7.04 (d, 2H), 4.23 - 4.17 (m, 4H), 4.06 - 3.98 (m, 6H), 3.94 - 3.43 (m, 27H), 1.80 (m, 2H), 1.52 - 1.26 (m, 40H), 0.90 (t, 3H). ^{13}C NMR (MeOD-d$_4$, 125 MHz) δ 161.6, 147.2, 146.8, 124.1, 124.0, 116.1, 114.5, 109.2, 78.8, 74.8, 72.2, 69.4, 68.1, 66.3, 31.8, 29.5, 29.4, 29.2, 29.1, 25.9, 24.4, 13.2. Exact mass calculated for $C_{56}H_{90}N_2O_{16}Na_1^+$ ([M+Na]$^+$): 1069.6183; found: 1069.6257.

[pG2]azoC$_{12}$ (n = 12): ^{1}H NMR (MeOD-d$_4$, 500 MHz) δ 7.96 - 7.90 (m, 4H), 7.23 - 7.19 (m, 2H), 7.12 - 7.07 (m, 2H), 4.79 - 4.72 (m, 1H), 4.36 - 3.50 (m, 36H), 1.89 - 1.82 (m, 2H), 1.58 - 1.50 (m, 2H), 1.47 - 1.35 (m, 40H), 0.97 (t, 3H). ^{13}C NMR (MeOD-d$_4$, 125 MHz) δ 162.8, 161.9, 148.4, 148.1, 125.4, 117.4, 115.8, 110.4, 80.1, 76.1, 76.0, 73.5, 72.5, 70.7, 69.4, 67.6, 30.7, 27.3, 27.2, 27.1, 25.9, 25.8, 25.7, 14.6. Exact mass calculated for $C_{57}H_{92}N_2O_{16}Na_1^+$ ([M+Na]$^+$): 1083.6339; found: 1083.6363.

General Procedure for the Synthesis of [G2]azoC$_n$ (n = 8 – 12)

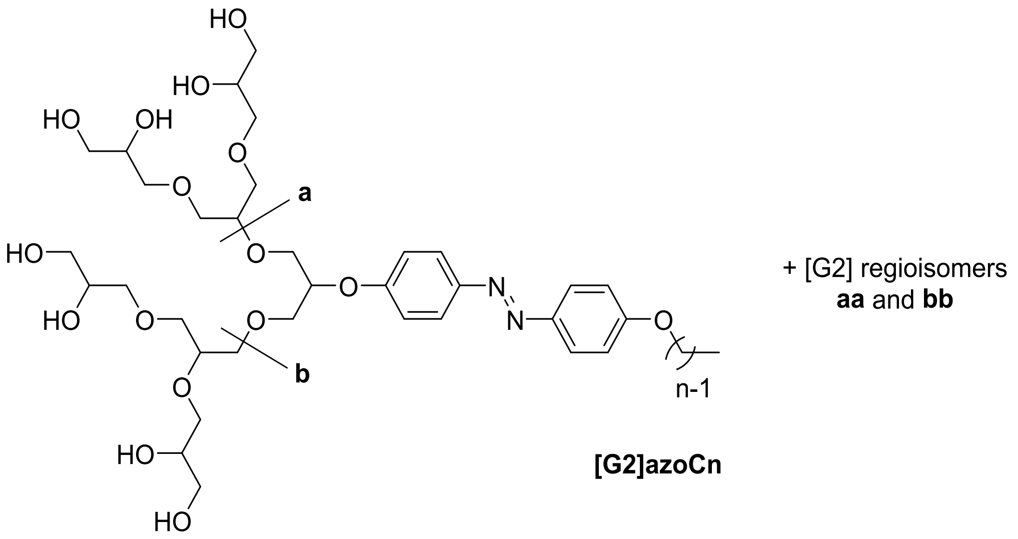

The desired [pG2]azoC$_n$ derivative was dissolved in a mixture of DMC (1.5 mL) and MeOH (1.5 mL) and HCl was added (20 µL, 37%). The mixture was stirred at room temperature for 18 hours and solvent was removed under reduced pressure. The raw product was dissolved in a mixture of ACN (2.5 mL) and H_2O (2.5 mL) and passed through a syringe filter (0.22 µM, RC). Subsequent RP HPLC purification (ACN/H_2O, 7:3 $\rightarrow$ 1:0) gave the desired product as orange-coloured wax (n = 8, 89 %; n = 9, 80 %; n = 10, 72 %; n = 11, 72 %; n = 12, 74 %).

[G2]azoC$_8$ (n = 8): ^{1}H NMR (MeOD-d$_4$, 700 MHz) δ 7.87 - 7.83 (m, 4H), 7.18 - 7.14 (m, 2H), 7.04 - 7.02 (m, 2H), 4.74 - 4.69 (m, 1H), 4.04 (t, 2H), 3.97 - 3.45 (m, 34H), 1.82 - 1.77 (m, 2H), 1.52 - 1.46 (m, 2H), 1.41 - 1.28 (m, 8H), 0.91 (t, 3H). ^{13}C NMR (MeOD-d$_4$, 175 MHz) δ 162.9, 161.8, 148.5, 148.1, 125.4, 125.3, 117.4, 115.8, 80.1, 78.4, 74.0, 72.4, 72.3, 72.2, 70.6, 69.4, 64.4, 33.0, 30.5, 30.4, 30.3, 27.1, 23.7, 14.4. Exact mass calculated for $C_{41}H_{68}N_2O_{16}Na_1^+$ ([M+Na]$^+$): 867.4461; found: 867.4505.

[G2]azoC$_9$ (n = 9): ^{1}H NMR (MeOD-d$_4$, 700 MHz) δ 7.83 - 7.80 (m, 4H), 7.14 - 7.11 (m, 2H), 7.01 - 6.99 (m, 2H), 4.70 - 4.65 (m, 1H), 4.01 (t, 2H), 3.94 - 3.41 (m, 34H), 1.79 - 1.74 (m, 2H), 1.48 - 1.43 (m, 2H), 1.37 - 1.23 (m, 10H), 0.87 (t, 3H). ^{13}C NMR (MeOD-d$_4$, 175 MHz) δ 162.9, 161.8, 148.5, 148.1, 125.4, 125.3, 117.4, 115.8, 80.1, 74.0, 73.9, 72.3, 72.2, 70.6, 69.4, 64.5, 33.0, 30.7, 30.5, 30.4, 30.3, 23.7, 14.4. m/z calcd for $C_{42}H_{70}N_2O_{16}Na_1^+$ ([M+Na]$^+$): 881.4618; found: 881.4675.

[G2]azoC$_{10}$ (n = 10): ^{1}H NMR (MeOD-d$_4$, 700 MHz) δ 7.87 - 7.84 (m, 4H), 7.18 - 7.15 (m, 2H), 7.06 - 7.03 (m, 2H), 4.74 - 4.70 (m, 1H), 4.07 (t, 2H), 3.99 - 3.46 (m, 34H), 1.84 - 1.80 (m, 2H), 1.54 - 1.49 (m, 2H), 1.43 - 1.28 (m, 12H), 0.91 (t, 3H). ^{13}C NMR (MeOD-d$_4$, 175 MHz) δ 161.5, 160.4, 147.2, 146.8, 123.9, 116.0, 114.4, 78.8, 77.6, 77.1, 72.6, 71.0, 70.8, 69.2, 68.0, 63.1, 31.7, 29.3, 29.1, 29.0, 25.7, 22.3, 13.0. Exact mass calculated for $C_{43}H_{72}N_2O_{16}Na_1^+$ ([M+Na]+): 895.4774; found: 895.4808.

[G2]azoC$_{11}$ (n = 11): ^{1}H NMR (MeOD-d$_4$, 700 MHz) δ 7.86 - 7.82 (m, 4H), 7.18 - 7.14 (m, 2H), 7.05 - 7.02 (m, 2H), 4.77 - 4.71 (m, 1H), 4.05 (t, 2H), 3.99 - 3.45 (m, 34H), 1.82 - 1.76 (m, 2H), 1.52 - 1.46 (m, 2H), 1.41 - 1.24 (m, 14H), 0.90 (t, 3H). ^{13}C NMR (MeOD-d$_4$, 175 MHz) δ 162.9, 161.6, 148.5, 148.1, 125.4, 125.3, 117.3, 115.8, 79.8, 78.3, 74.1, 72.4, 71.9, 70.4, 69.4, 64.5, 33.0, 30.7, 30.5, 30.4, 27.1, 23.7, 14.4. Exact mass calculated for $C_{44}H_{74}N_2O_{16}Na_1^+$ ([M+Na]$^+$): 909.4931; found: 909.4878.

[G2]azoC$_{12}$ (n = 12): ^{1}H NMR (MeOD-d$_4$, 700 MHz) δ 7.86 - 7.82 (m, 4H), 7.16 - 7.13 (m, 2H), 7.03 - 7.01 (m, 2H), 4.73 - 4.67 (m, 1H), 4.03 (t, 2H), 3.95 - 3.45 (m, 34H), 1.81 - 1.76 (m, 2H), 1.50 - 1.45 (m, 2H), 1.39 - 1.24 (m, 16H), 0.89 (t, 3H). ^{13}C NMR (MeOD-d$_4$, 175 MHz) δ 162.8, 161.8, 148.5, 148.1, 125.4, 125.3, 117.4, 115.8, 80.1, 74.0, 73.9, 72.3, 72.1, 70.5, 69.4, 64.4, 33.0, 30.7, 30.7, 30.5, 30.3, 27.1, 23.7, 14.4. Exact mass calculated for $C_{45}H_{76}N_2O_{16}Na_1^+$ ([M+Na]$^+$): 923.5087; found: 923.5143.

Quantification of Isomers in Solution

The *cis/trans* equilibrium of **3**, **6**, **8**, and **10** was monitored in solution after full equilibration under irradiation at 366 nm either by UV/VIS spectroscopy and/or RP HPLC analysis. The applied light source is a UV/VIS lamp from CAMAG and was described previously in more detail.[2] The time required to achieve full saturation of *cis* under irradiation at 366 nm was determined as shown exemplarily in Figure S1. Isomer ratios are summarized in Table S1 – 2.

UV/VIS Spectroscopy. For UV/VIS measurements, a LAMBDA 950 UV/Vis/NIR spectrophotometer from PerkinElmer and standard disposable PMMA UV/VIS cuvettes with a path length of 1 cm from Plastibrand were used. The maximal reduction in intensity of the absorption maximum of *trans* (ΔI_{A356}) was obtained after an exposure time of 120 s. Data were analysed with Origin V9.1. The amount of *cis* was calculated from the irradiation time-dependent (*t*) decrease in intensity (ΔI) of the absorption maximum of *trans* (A_{max} = 356 nm) using the following equation:

$$cis\ (\%) = \ \Delta I_{A356} \cdot 100\% = [I_{A356}(t = 0\ s) - \ I_{A356}(t = 120\ s)]\ \cdot 100\%$$

HPLC Analysis. RP HPLC analysis was performed with a system from VWR Hitachi, which was equipped with an organizer, pump 5160, auto sampler 5260, column oven 5310, and a diode array detector 5430. The applied method was: water/acetonitrile 1 ⟶ 99% *v/v*, run duration: 14 min, flow rate: 1 mL/min. Briefly, *cis* and *trans* were separated on an RP C18 column (Purospher® STAR, RP-18 endcapped, 2µm) prior to their quantification with a UV/VIS detector. The detection wavelength was adjusted to the isosbestic point at which both isomers exhibit similar extinction coefficients (315 nm).[3] Data analysis was executed with ChromeGate Client Viewer (*v*.3.3.2) from Knauer.

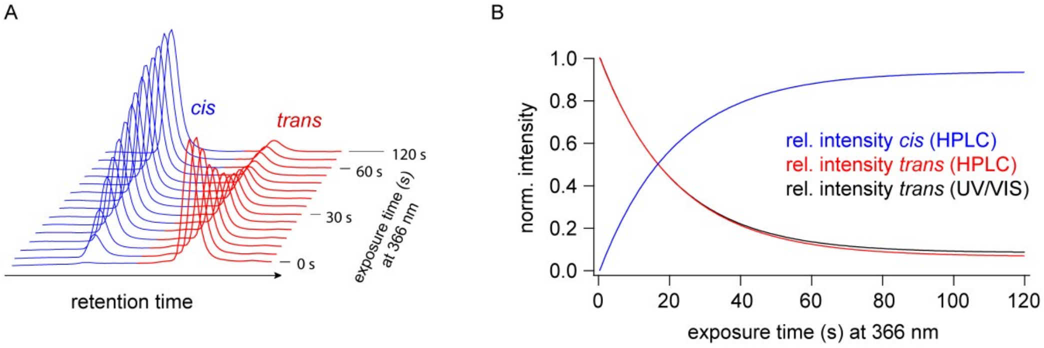

Fig. S1. Determining the irradiation time required to achieve maximal saturation of *cis* OGBAs under irradiation at 366 nm. OGBA **1** was dissolved exemplarily in a mixture of water and methanol (v:v, 1:1, c ~ 50 µM, V ~ 3 mL) and transferred into standard disposable PMMA UV/VIS cuvettes with a path length of 1 cm (Plastibrand). The cuvette was irradiated at 366 nm for five seconds and small aliquots (50 µL) of were subjected to HPLC analysis. (A) The peaks obtained at shorter retention time were assigned as *cis* and the peaks at longer retention time were assigned as *trans.* The procedure was repeated until the *trans* to *cis* conversion was found to be completed. (B) Peak area values were extracted from the chromatograms, normalized to the initial intensity of *trans* (exposure time = 0 s), and plotted against the exposure time. Data were fitted to exponential functions, which show that full saturation of *cis* is obtained after 120 s under irradiation at 366 nm. Relative depletion in UV/VIS intensity at the absorption maximum of *trans* (A_{max} = 356 nm) show a similar result. Previous studies proved that similar isomerization kinetics can be assumed for OGBAs **2 – 5**.[3]

Table S1. Comparison of *cis/trans* ratios obtained from OGBA **3** upon irradiation at 366 nm by solution techniques and IM-MS. Ratios obtained by UV/VIS and HPLC experiments in solution were exclusively reproduced by DT IM-MS analysis of $[M+2H]^{2+}$ ions. Similar *cis/trans* ratios are highlighted in bold.

**Experiment**	OGBA **3** *cis/trans*
**UV/VIS**	**88/12**
**HPLC**	**91/09**
Synapt $[M+2H]^{2+}$	35/65
**DT $[M+2H]^{2+}$**	**89/11**
Synapt $[M+2Na]^{2+}$	80/20
DT $[M+2Na]^{2+}$	78/22

Table S2. Comparison of *cis/trans* ratios obtained from OGDs **6**, **8**, and **10** upon irradiation at 366 nm by UV/VIS and IM-MS. Ratios obtained by UV/VIS in solution were exclusively reproduced by DT IM-MS analysis of $[M+Na]^{+}$ ions. Similar *cis/trans* ratios are highlighted in bold.

**Experiment**	OGD **6** *cis/trans*	OGD **8** *cis/trans*	OGD **10** *cis/trans*
**UV/VIS**	**82/18**	**78/22**	**86/14**
DT $[M+H]^{+}$	97/3	90/10	96/4
**DT $[M+Na]^{2+}$**	**83/17**	**82/18**	**84/16**

Ion Mobility-Mass Spectrometry

IM-MS experiments were executed either on a commercially available travelling-wave quadrupole/ion mobility time-of-flight mass spectrometer, Synapt G2-S HDMS (Waters Corporation)[6] or a linear drift tube (DT) instrument (homebuilt, Fig S5).[7] Both instruments were equipped with a nano electrospray ionization (nESI) source and mass-calibrated using a solution of caesium iodide (c = 100 mg/mL).

Preparation of nESI capillaries. The nESI capillaries were produced from borosilicate capillaries (diameter: 1.0 mm) using a needle puller (Flaming/Brown MicropipetteP-1000, Sutter Instrument Company, Novato, USA). The capillaries were coated with a Pt/Pd (80/20) mixture using a sputter coater (Sputter Coater HR 208, Cressington, Dortmund, Germany). The procedure was adopted from Göth *et al.*[8]

Synapt. Instrument parameters were: capillary voltage (0.7 kV), sample cone (60 V), source offset (20 V), cone gas (0 L/h), trap collision energy (4 V), trap DC bias (50 V), IM gas (helium), IM gas flow (90 mL/min), IM drift voltage (50 V), ion transfer stage pressure ($2.41 \cdot 10^{-2}$ mbar), trap pressure ($2.22 \cdot 10^{-2}$ mbar).

DT Instrument. Instrumental parameters on the DT instrument were: capillary voltage (0.8 kV), V_{end} (160 V), IM gas (helium), pressure inside the drift tubes (4.86 mbar), temperature (23.5 °C), length of drift tube (80.55 cm). A more detailed description of the instrument has been reported previously.[7]

Quantification of Isomers by IM-MS. Peak area values were extracted from ATDs of doubly charged *cis* and *trans* **3** or singly charged *cis* and *trans* **6**, **8**, **10**. The ratio between both area values was taken as isomer ratio. Data are summarized in Table S1 – S2.

CCS calculation. Drift times were converted into CCS values using the Mason Schamp equation as described previously:[7, 9] Briefly, ions were generated by a nanoelectrospray ionisation (nESI) source (positive ion mode) and transferred into an ion funnel from which the ions are pulsed into the drift tube region of the instrument (Fig. S5). Guided by a weak electric field the ions traversed the drift tubes, which were filled with helium gas. The time required for the ions to pass the drift tube region depends on their mobility, which in turn is defined by the molecules' overall shape and charge. Subsequently, the ions were guided by a second ion funnel into high vacuum where the ions were selected according to their *mass-to-charge* ratio (*m/z*) using a quadrupole mass analyser. The arrival time distributions (ATDs) of the ions are recorded by measuring the time that ions of a specific *m/z* need to traverse the drift region. The time which corresponds to the intensity maximum of the ATD was taken as the drift time (t_D). The t_D values were determined for different drift voltages (V_D) and plotted against $1/V_D$. The data were fitted to a linear function. The mobility of the ions was calculated from the slope of the linear fit. Finally, the collision cross section (CCS) was determined by using the Mason-Schamp equation.

Theoretical CCS calculation. Conformational search and optimization of individual conformers of OGBA **1** was achieved using the MM2 force field as implemented in ChemBio3D v14.0 (PerkinElmer). Theoretical CCS values were calculated using the projection approximation algorithm.[10]

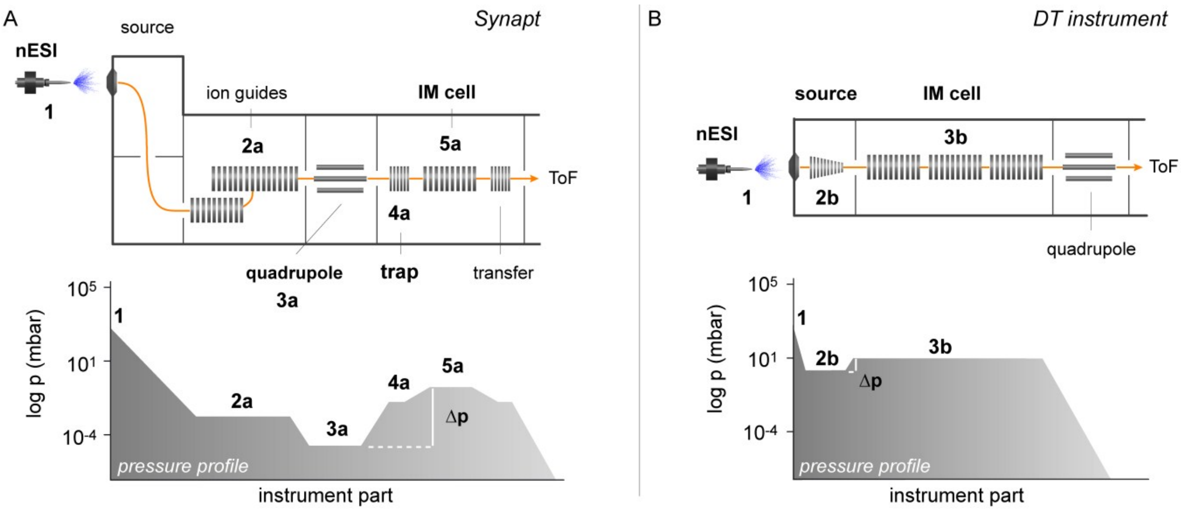

Fig. S2. Comparing instrument setups of (A) a commercially available Synapt and (B) a homebuilt drift tube (DT) instrument. The pressure gap between quadrupole (**3a**) and IM cell (**5a**) in the Synapt is larger than the pressure gap between source (**2b**) and IM cell (**3b**) in the DT instrument. In case of the DT instrument, a lower acceleration voltage is required to transmit ions into the IM cell, which causes less thermal heating and "softer" transmission conditions for the ions.

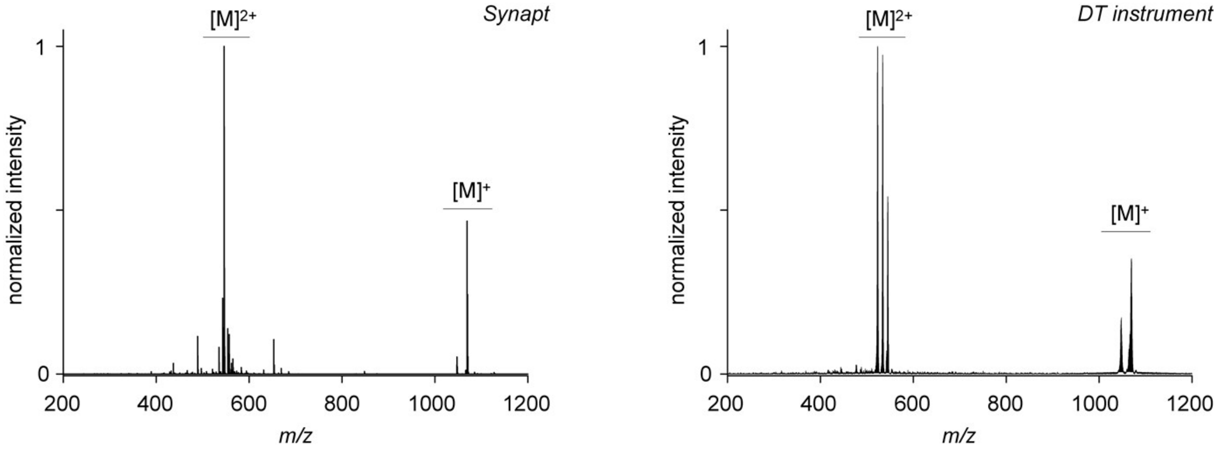

Fig. S3. Mass spectra of OGBA **3** obtained from a Synapt G2 S HDMS instrument (left) and a homebuilt DT instrument (right). The spectra reveal singly charged ions $[M]^{+}$ and doubly charged ions $[M]^{2+}$. Sample conditions: OGBA **3** was dissolved in a mixture of water and methanol (v:v, 1:1, c ~ 10µM).

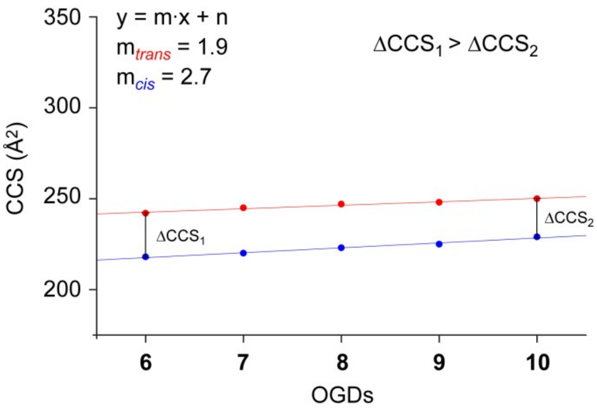

Fig. S4. CCS data obtained from singly sodiated ions of *cis* and *trans* **6** – **10** were fitted to linear functions (y = m·x + n). The difference in CCS between *cis* and *trans* (ΔCCS) decreases in the direction of **6** → **10**. This underlines that increasing the size of the hydrophobic backbone reduces the impact of the light-driven conformational change on the overall molecular shape.

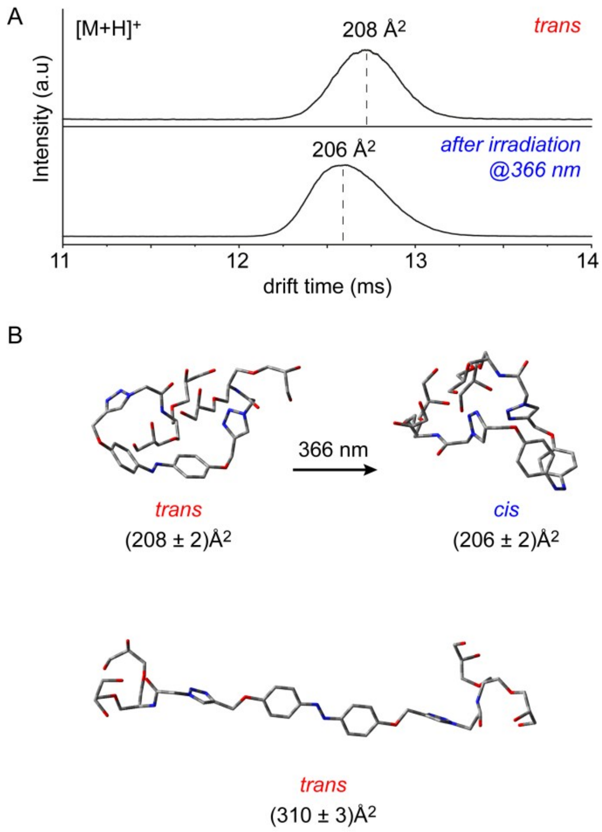

Fig. S5. Investigating the gas-phase structure of OGBA **1** by IM-MS and molecular dynamics. (A) IM-MS analysis of singly protonated **1** $[M+H]^+$ using the DT instrument revealed a small shift to lower drift times/CCS values after full equilibration of the analyte under irradiation at 366 nm in solution. (B) Similar theoretical CCS values are obtained from conformers in which both head groups are collapsed. As a control, the theoretical CCS value from an extended conformer of *trans* **1** is shown. Conformer search was done with ChemBio3D Ultra v14.0. Theoretical CCS values were calculated using the projection approximation algorithm.[10]

Gas-Phase Infrared Spectroscopy

To obtain IR spectra in the gas phase, an infrared laser of high intensity was applied to allow for the absorption of multiple photons at IR-active sites of ions. This resulted in increased internal energies and dissociation of covalent bonds within ions. This way of obtaining an IR spectrum is called: IR multiple photon dissociation (IRMPD) experiment.[7] Here, the Fritz Haber Institute free electron laser (FEL) was coupled to the DT IM-MS instrument, which enabled spectroscopic investigation of drift-time selected species. Subsequent to the drift tubes, ions within a narrow drift-time window were selected using the quadrupoles and irradiated by a pulse of intense IR light. Subsequent fragment analysis occurred by means of TOF MS. An action spectrum was recorded by monitoring the intensity of the precursor ion signal and the corresponding fragment signals as a function of the laser wavelength.

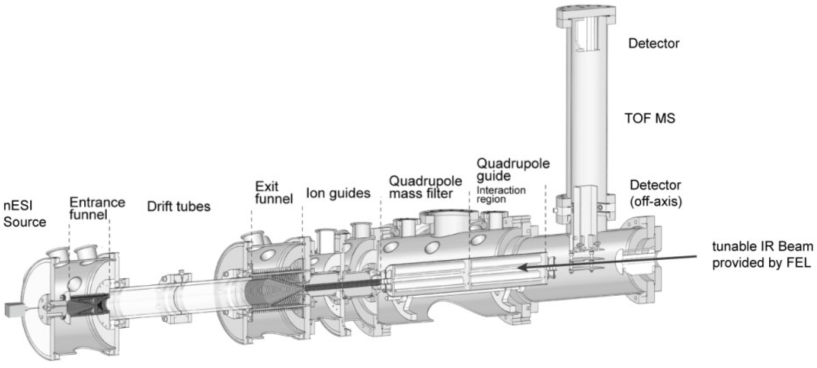

Fig. S6. Instrument setup of the utilized DT instrument.[7]

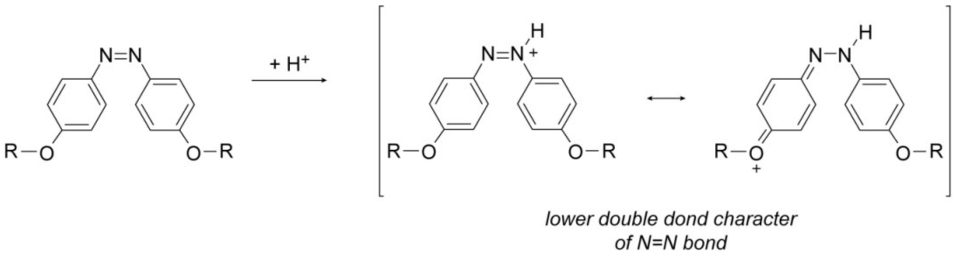

Fig. S7. Proposed structural formulas that explain how protonation in singly charged ions of **1** $[M+H]^+$ lowers the double bond character of the *cis* N=N bond. Mesomeric formulas underline that the of alkoxy substituents in *para* position to the N=N bond contribute to the lower double bond character due to their electron donating properties (+M effect). The full structure of abbreviated side chains (R) is shown in Figure 1 of the manuscript.

Table S3. Gas-phase basicity values (ΔG) of functional groups in OGBA **1**. Values were estimated from data published for chemically similar functional groups – see Hunter *et al.*[11] and Abboud *et al.*[12]

Functional Group	ΔG (kJ/mol)
hydroxyl group (prim.)	756 – 764[a]
hydroxyl group (sec.)	784[b]
ether	794[c]
amide	820[d]
triazole	925[e]
trans N=N bond (dimethoxy-substituted azobenzene)	824[*]
cis N=N bond (dimethoxy-substituted azobenzene)	885[*]

[a]*estimated from 1-propanol – see Hunter et al.*[11]
[b]*estimated from 2-butanol – see Hunter et al.*[11]
[c]*estimated from tetrahydrofuran – see Hunter et al.*[11]
[d]*estimated from N-methylformamide – see Hunter et al.*[11]
[e]*estimated from 1-Methyl-4-phenyl-1,2,3-triazole – see Abboud et al.*[12]
[*]*estimated by plotting protein charge reduction (%) as a function of gas-phase basicity of the detergents' linker between head group and tail – see Fig. S12*

Membrane Protein Solubilisation

Purification and preparation of AmtB for native MS. AmtB was overexpressed as an AmtB-MBP construct in *E. coli* and purified as described before.[13] The protein-containing membranes were solubilized at 4 °C in resuspension buffer (100 mM NaCl, 20 mM Tris, 5 mM BME, 20% *v/v* glycerol, pH = 7.4) supplemented with *n*-octyl-ß-D-glucoside (200 mM). The supernatant was clarified by centrifugation (20000 x g, 25 min, 4°C), filtered (0.22 µm syringe filter), and loaded on a HisTrap-HP column (5 mL, GE healthcare) that was equilibrated with DDM-containing immobilized metal ion affinity chromatography (IMAC) wash buffer (200 mM NaCl, 50 mM Tris, 10% *v/v* glycerol, 2x*cac* DDM, 20 mM imidazole, pH = 7.4). The column was washed with 40 mL of IMAC wash buffer (200 mM NaCl, 50 mM Tris, 10% *v/v* glycerol, 1w% OG, 20 mM imidazole, pH = 7.4) and then washed with DDM-containing IMAC wash buffer (200 mM NaCl, 50 mM Tris, 10% *v/v* glycerol, 2x*cac* DDM, 20 mM imidazole, pH = 7.4) until a steady baseline was observed. The protein was eluted with a linear gradient over two column volumes to 100% of DDM-containing IMAC elute buffer (100 mM sodium chloride, 50 mM Tris, 10% *v/v* glycerol, 2x*cac* DDM, 500 mM imidazole, pH 7.4). The fractions were collected, supplemented with His-tagged TEV (1 mg TEV per 3 – 5 mg membrane protein) and transferred into a Slide-A-Lyzer dialysis cassette (MWCO = 3.5 kDa, Thermo Fisher Scientific). The mixture was dialyzed for 12 hours at 4 °C against dialysis buffer (2 L of 150 mM NaCl, 50 mM Tris, 10% *v/v* glycerol, 2x*cac* DDM, 20 mM imidazole, pH = 7.4). The mixture was passed over a HisTrap-HP column and the protein was eluted with dialysis buffer. The protein was concentrated using Amicon-Ultra centrifugal filter units (MWCO = 100 kDa) to a final concentration of above 40 µM.

The detergent exchange into *trans* **6** was done over a 3 mL size exclusion chromatography (SEC) column (Superdex 200 10/300GL column, product number: 17-5175-01) using an ÄKTA setup. The column was equilibrated with ammonium acetate buffer (NH_4OAc, 200 mM, pH = 6.8) containing 2x*cac* of *trans* **6**. For further information about the *cac* of **6**, before and after irradiation at 366 nm, see Fig S9. All transparent tubes and components of the ÄKTA setup were covered with aluminum foil. A 50 µL aliquot of AmtB (c > 40 µM) was injected into the column and AmtB was eluted with a flow rate of 0.2 mL/min over 1.5 column volumes. Fractions were collected, concentrated using Amicon-Ultra centrifugal filter units (MWCO = 100 kDa), and analyzed with a Synapt G1 instrument (operated in MS mode) using the following experimental parameters: capillary voltage (1.5 kV), cone voltage (120 V), source temperature (25 °C), and trap collision energy (190 V). Sample cone, extraction cone, trap DC bias, and source pressure were optimized for maximum ion intensity of the AmtB trimer. The protein sample was analysed before and after irradiation at 366 nm using similar instrumental parameters. The mass spectra were acquired and analysed using MassLynx and plotted using Origin V9.1.

CD Spectroscopy. AmtB solubilized in DDM (concentration of the AmtB trimer = 4.6 µM) was transferred into ammonium bicarbonate buffer (NH_5CO_3, 100 mM, pH = 8) containing DDM, *trans* **6**, or a *cis/trans* mixture of **6** obtained after full conversion of the isomer mixture under irradiation at 366 nm. The protein solutions were loaded into cuvettes (Quartz Suprasil, volume = 300 µL, layer thickness = 1 mm) and analyzed using a CD spectrometer (Chirasan, USA). The setup was flushed with nitrogen gas overnight and the lamp as well as the thermostat was turned on 30 min before the first measurement. The experimental parameters were as follows: final concentration of the AmtB trimer (0.14 µM), temperature (22.5 °C), wavelength range (190 – 260 nm), step size (1 nm), scan speed (0.5s/nm), bandwidth (1 nm), and repeats per sample (4). Detergent-containing NH_5CO_3 buffers were used as blanks. Data were acquired with Pro-Data Chirascan V4.5 and analyzed with Origin V9.1 (see Fig.S8). The average intensity of four scans was plotted against the wavelength (Fig. S8).

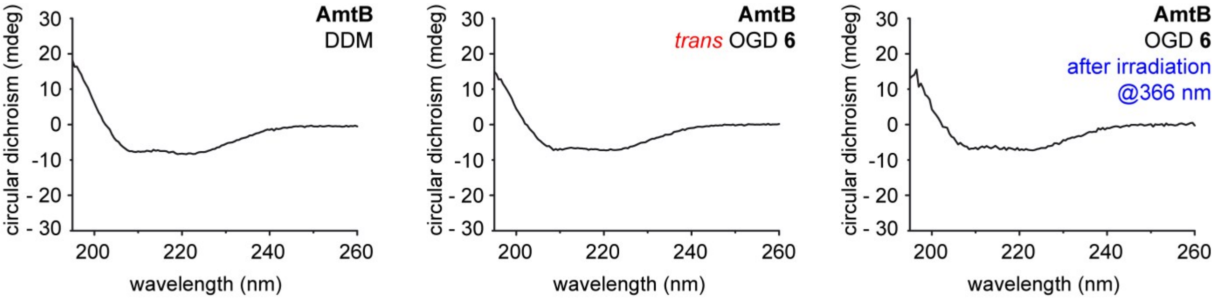

Fig. S8. Investigating the native secondary structure of AmtB in solution. CD Spectra of AmtB in NH_5CO_3 buffer (100 mM) similar alpha-helical content in DDM, *trans* **6**, and after irradiation at 366 nm. Spectra were recorded at concentration of 0.14 µM for the AmtB trimer. DDM, *trans* **6**, and the *cis/trans* mixture of **6** after irradiation at 366 nm can preserve the native fold of AmtB in solution.

Dynamic Light Scattering. The *cac* of OGD **6** was determined by dynamic light scattering (DLS). A dilution series was prepared in MilliQ water with concentrations of **6** between 10^{-8} and 10^{-2} mol·L^{-1}. The samples were filtered (0.22 µm, RC) and equilibrated in the dark for at least five days at room temperature (approximately 22 °C) to ensure a maximum saturation of the *trans* form. The samples were analysed in cuvettes (Quartz Suprasil, width x length: 2 mm x 10 mm) using a Zetasizer Nano-ZS ZEN3600 (Malvern, UK). We found no evidence that the laser irradiation of this Zetasizer instrument (red laser, 632.8 nm) is affecting the *cis/trans* ratio of our amphiphiles in solution.[3] The instrumental parameters were as follows: material (polystyrene latex), dispersant (water), sample viscosity parameters (use dispersant viscosity as sample viscosity), temperature (22.5 °C), equilibration time (120 s), cell type (quartz cuvettes), measurement angle (173° backscatter), measurement duration (manual), number of runs (11), run duration (10 s), number of measurements (3), delay between the measurements (0 s), data processing (general purpose, normal resolution). The derived count rate values obtained from three measurements per concentration were averaged. The unit of the derived count rate is kilo counts per second (kcps). The logarithm of the derived count rate was plotted against the logarithm of the concentration. The double logarithmic plots showed two characteristic regions: (1) a flat region with low count rates at lower concentrations of **6** and (2) a linear growth of the count rate at higher concentrations of **6**. Both regions were fitted to linear functions and the intersection was taken as the *cac* value (Fig. S9).[14] A monomodal average size distribution of particles was obtained above the *cac*, before and after irradiation at 366 nm (volume%). The diffusion coefficients related to the obtained intensity maxima (65.4 µm·s^{-2}) and the calculated hydrodynamic diameters (5.61 nm) indicate that both *trans* **6** and its isomer mixture after irradiation at 366 nm are forming micelles above a concentration of 250 µM (Fig. S9).

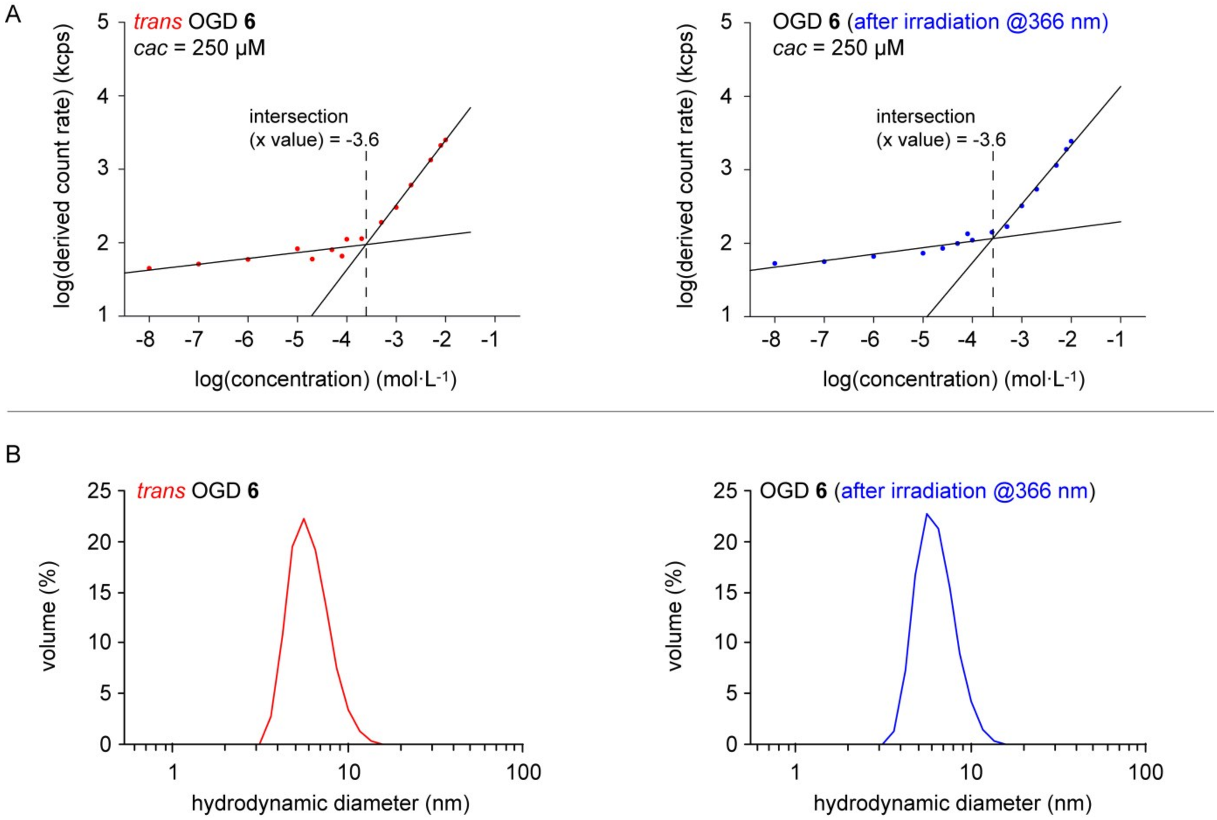

Fig. S9. Investigating the aggregation behaviour of OGD **6** in water using DLS. (A) Concentration-dependent analysis of the derived count rate revealed similar *cac* values for **6**, before and after irradiation at 366 nm. (B) The diffusion coefficients of the aggregates formed (65.4 µm·s^{-2}) as well as the calculated hydrodynamic diameters (5.61 nm) match with those that have been previously obtained from micelle-forming OGDs.[15] The DLS data therefore indicate that both *trans* **6** as well as the isomer mixture obtained after irradiation at 366 nm are forming micelles.

Protein Mass Spectrometry

Preparation of Protein-Detergent Complexes. The protein-detergent complexes (PDCs) formed by OGDs and ß-lactoglobulin (BLG) were prepared using a previously published procedure.[16] Briefly, BLG was dissolved in NH_4OAc buffer (10 mM) to a final concentration of 1 mg/mL. The protein sample (500 µL) was loaded into an Amicon-Ultra 0.5 mL centrifugal device (MWCO = 10 kDa, Merk Millipore, Germany). The samples were concentrated (14000 x g, 10 min), diluted with NH_4OAc buffer to a final volume of 500 µL, and concentrated again. This procedure was repeated five times. The final protein concentration was determined by UV/VIS spectroscopy using the extinction coefficient of BLG (17600 $M^{-1}\cdot cm^{-1}$).[17] Equimolar protein-OGD mixtures were prepared (1:1, 50 µM) by dilution in order to generate a 1:1 stoichiometry. The samples were subjected to nESI-MS analysis as described below.

MS Analysis of Protein-Detergent Complexes. Mass spectra of BLG-OGD complexes were obtained in positive ionization mode using a modified Ultima high-mass quadrupole time-of-flight mass spectrometer (Waters Micromass, Manchester, UK). The instrument was equipped with a Z-spray nESI source. Instrument conditions that led to the obtainment of intense PDC signals were adopted from a previously reported procedure:[16] capillary voltage (1.2 kV), cone voltage (35 V), RF lens (50 V), collision gas pressure (P_{argon} ~ $5\cdot10^{-3}$ mbar), collision voltage (2 V), and *m/z* range (200 – 5000). For tandem mass spectrometry (MS/MS) experiments, the ions were selected according to their m/z and forced to dissociation by increasing the collision voltage to 60V. Quadrupole settings were: LM res (3.3 V) and HM res (15 V). Data were acquired with MassLynx V4.1 and analysed by means of Origin V9.1.

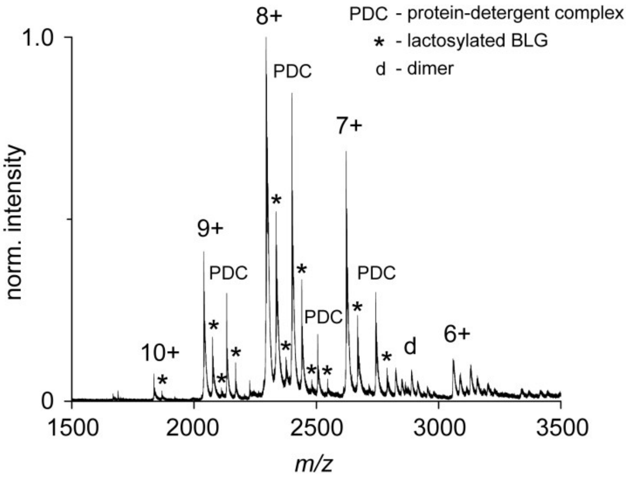

Fig. S10. Mass spectrum obtained from an equimolar mixture of BLG and *trans* **6** (50 µM) in ammonium acetate buffer (10 mM). Protein charge states from 6+ to 10+ are obtained. The spectrum shows the *apo* form of BLG, protein-detergent complexes (PDCs), and lactosylated forms of BLG.

Remarks to the Design of [G2]etherC18, [G2]amideC18, and [G2]triazoleC18. For the formation PDCs it is important that both protein and detergent are sufficiently hydrophobic. A substitution of azobenzene in OGD **6** with alternative linker groups, such as ether, amide, or triazole, would lead to a detergent with a [G2] head group and a comparatively short hydrophobic tail, *e.g.*, C8. Such a detergent would be very hydrophilic. In order to increase the hydrophobicity of the detergent, we added ten more carbon atoms to the hydrophobic chain. We assume that the length of the hydrophobic chain is important for the formation of PDCs in solution and that it has a negligible impact on the basicity of the detergent compared to that of the alternative linker groups, such as ether, amide, and triazole.

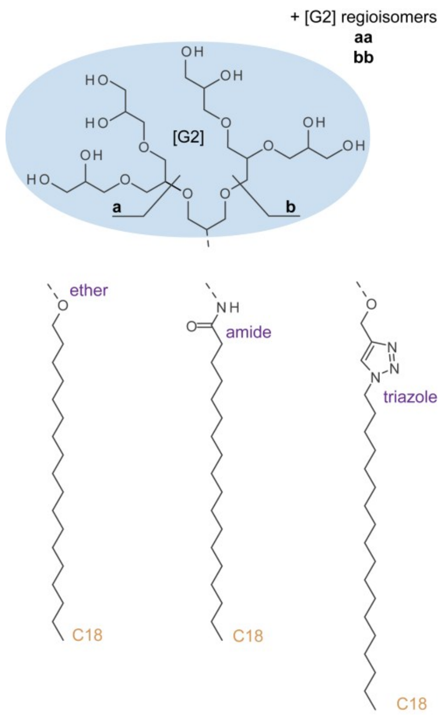

Fig S11. Structure of OGDs in which azobenzene was substituted for alternative linker groups (ether, amide, triazole).

Estimating the Gas-Phase Basicity of OGD 6. Mass spectra obtained upon dissociation of PDCs show fractions of non-charge-reduced BGL ($[P]^{8+}$), charge-reduced BLG ($[P]^{7+}$), and singly-charged detergent ions $[D]^{1+}$ (see Figure 5 in manuscript). The loss of neutral detergent molecules $[D]^{0\pm}$ is not detected under the experiment conditions employed. The PDC dissociation products are summarized as follows:

$$[PDC]^{8+} \rightarrow [P]^{8+} + [P]^{7+} + [D]^{1+} + [D]^{0\pm}$$

To estimate the gas-phase basicity of OGD **6** we assumed that the fractions of charge-reduced and non-charge-reduced BLG ions $\left(\frac{[P]^{7+}}{[P]^{8+}}\right)$ is proportional to the gas-phase basicity of the detergents' linker between head and tail (ΔG_{linker}):

$$fraction\ of\ charge-reduced\ BLG\ (\%) = \left(\frac{[P]^{7+}}{[P]^{8+}}\right) \cdot 100\% \sim \Delta G_{linker}$$

The fractions of charge-reduced BLG (%) obtained from OGDs in which azobenzene was substituted for alternative linker groups, such as ether, amide, or triazole, have recently been reported elsewhere.[16] Here, the fractions of charge-reduced BLG (%) were plotted against the gas-phase basicity of the detergents' linker groups of [G2]etherC18, [G2]amideC18, and [G2]triazoleC18. The average gas-phase basicity values of OGD **6**, before and after irradiation at 366 nm, were extrapolated by assuming a linear correlation between the fraction of charge-reduced BLG (%) and gas-phase basicity of the detergents' linker groups under the experimental conditions employed (Fig.12).

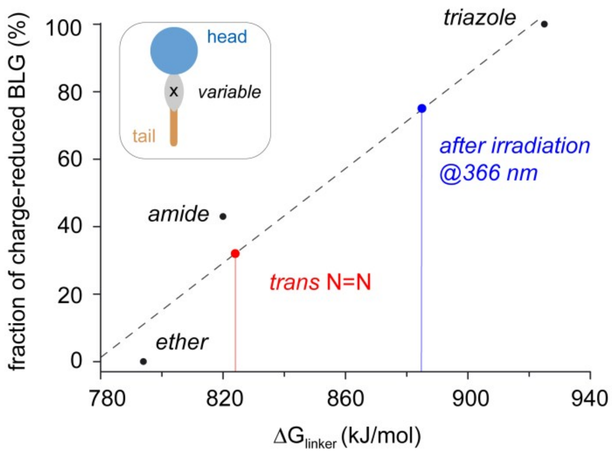

Fig. 12. Estimating the average gas-phase basicity of OGD **6**, before and after irradiation of the sample solution at 366 nm. The fraction of charge-reduced BLG (%) was plotted against the gas-phase basicity values of linker groups between head group and tail of [G2]etherC18, [G2]amideC18, and [G2]triazoleC18. Extrapolating the gas-phase basicity values of **6** indicates that the gas-phase basicity went up by 60 kJ/mol after irradiation at 366 nm. This is in line with the obtained increase in BLG charge reduction upon PDC dissociation (see Figure 5 in manuscript).

Literature

1. C. Kördel, C. S. Popeney and R. Haag, *Chem. Commun.*, 2011, **47**, 6584-6586.
2. L. H. Urner, B. N. S. Thota, O. Nachtigall, S. Warnke, G. von Helden, R. Haag and K. Pagel, *Chem. Commun.*, 2015, **51**, 8801-8804.
3. L. H. Urner, B. Schade, M. Schulze, K. Folmert, R. Haag and K. Pagel, *ChemPhysChem*, 2019, **20**, 1690-1697.
4. O. Nachtigall, C. Kördel, L. H. Urner and R. Haag, *Angew. Chem. Int. Ed.*, 2014, **53**, 9669-9673.
5. M. Wyszogrodzka and R. Haag, *Chem. Eur. J.*, 2008, **14**, 9202 - 9214.
6. S. D. Pringle, K. Giles, J. L. Wildgoose, J. P. Williams, S. E. Slade, K. Thalassinos, R. H. Bateman, M. T. Bowers and J. H. Scrivens, *Int. J. Mass Spectrom.*, 2007, **261**, 1-12.
7. S. Warnke, J. Seo, J. Boschmans, F. Sobott, J. H. Scrivens, C. Bleiholder, M. T. Bowers, S. Gewinner, W. Schöllkopf, K. Pagel and G. von Helden, *J. Am. Chem. Soc.*, 2015, **137**, 4236-4242.
8. M. Göth, F. Lermyte, X. J. Schmitt, S. Warnke, G. von Helden, F. Sobott and K. Pagel, *Analyst*, 2016, **141**, 5502 - 5510.
9. H. V. Schröder, A. Mekic, H. Hupatz, S. Sobottka, F. Witte, L. H. Urner, M. Gaedke, K. Pagel, B. Sarkar, B. Paulus and C. A. Schalley, *Nanoscale*, 2018, **10**, 21425-21433.
10. G. von Helden, M. T. Hsu, N. Gotts and M. T. Bowers, *J. Phys. Chem.*, 1993, **97**, 8182-8192.
11. E. P. Hunter and S. G. Lias, *J. Phys. Chem. Ref. Data*, 1998, **27**, 413 - 656.
12. J.-L. M. Abboud, C. Foces-Foces, R. Notario, R. E. Trifinov, A. P. Volovodenko, V. A. Ostrovskii, I. Alkorta and J. Elguero, *Eur. J. Org. Chem.*, 2001, **2001**, 3013-3024.
13. A. Laganowsky, E. Reading, T. M. Allison, M. B. Ulmschneider, M. T. Degiacomi, A. J. Baldwin and C. V. Robinson, *Nature*, 2014, **510**, 172-175.
14. Y. Skhiri, P. Gruner, B. Semin, Q. Brosseau, D. Pekin, L. Mazutis, v. Goust, F. Kleinschmidt, A. El Harrak, J. B. Hutchison, E. Mayot, J.-F. Bartolo, A. D. Griffiths, V. Taly and J.-C. Baret, *Soft Matter*, 2012, **8**, 10618-10627.
15. B. N. S. Thota, H. v. Berlepsch, C. Böttcher and R. Haag, *Chem. Commun.*, 2015, **51**, 8648 - 8651.
16. L. H. Urner, Y. B. Maier, R. Haag and K. Pagel, *J. Am. Soc. Mass Spectrom.*, 2018, **30**, 174-180.
17. M. I. Viseu, T. I. Carvalho and S. M. B. Costa, *Biophys. J.*, 2004, **86**, 2392 - 2402.
